# Lapatinib and lapatinib plus trastuzumab therapy versus trastuzumab therapy for HER2 positive breast cancer patients: an updated systematic review and meta-analysis

**DOI:** 10.1186/s13643-022-02134-9

**Published:** 2022-12-10

**Authors:** Ye Yuan, Xumei Liu, Yi Cai, Wenyuan Li

**Affiliations:** Hospital of Chengdu University of Traditional Chinese Medicine, Chengdu, China

**Keywords:** Lapatinib, Trastuzumab, HER2 positive, Breast cancer, Randomized controlled trials, Meta-analysis, Systematic review

## Abstract

**Introduction:**

Trastuzumab, as the gold standard for HER2-positive BC treatment, was the first-line HER2 targeted drug. However, some studies reported patients benefited more from lapatinib and lapatinib plus trastuzumab therapy than standard trastuzumab therapy. This study presents an update of a systematic review and meta-analysis involving comparison of lapatinib and lapatinib plus trastuzumab therapy versus trastuzumab therapy.

**Aim:**

We determined whether trastuzumab plus lapatinib or lapatinib therapy is not inferior to trastuzumab therapy in HER2-positive breast cancer patients.

**Methods:**

Relevant trials were searched in CNKI, Wanfang, VIP, Sinomed, PubMed, Embase, and Cochrane CENTRAL databases from inception until October 25, 2021. Primary outcomes were OS, DFS/EFS, and PFS while secondary outcomes were pCR (ypT0/is ypN0), pCR (ypT0/is ypN0/+), ORR, DCR, rate of BCS, RFS, cardiac toxicities, and other toxicities.

**Results:**

Thirteen randomized controlled trials were included in this study. Trastuzumab combined with lapatinib therapy was found to be superior to standard trastuzumab therapy alone with regard to overall survival, disease-free survival/event-free survival, pathologic complete response (ypT0/is ypN0), pathologic complete response (ypT0/is ypN0/+), recurrence-free survival, higher incidences of diarrhea, and rash/skin toxicity. Lapatinib therapy was established to be inferior to trastuzumab therapy in overall survival, progression-free survival, disease-free survival/event-free survival, pathologic complete response (ypT0/is ypN0) and pathologic complete response (ypT0/is ypN0/+), diarrhea, and rash/skin toxicity and had a low incidence of left ventricular ejection fraction decline.

**Conclusions:**

The efficacy of trastuzumab combined with lapatinib therapy is superior to standard trastuzumab therapy alone; however, it has more non-cardiac grade III/IV toxicities. Moreover, the efficacy of lapatinib therapy is inferior to that of standard trastuzumab therapy alone.

**Supplementary Information:**

The online version contains supplementary material available at 10.1186/s13643-022-02134-9.

## Introduction

Globally, breast cancer (BC) is the most common cancer and is the leading cause of tumor-associated mortalities among women. It accounts for about 30% of female cancer incidences and 15% of all cancer deaths [1–3]. Incidence and mortality rates of BC have been consistently increasing, with about 0.5% and 0.7 per 100,000 annual growth, respectively [3, 4]. Moreover, as a heterogeneous disease, BC has four major molecular subtypes divided by gene expressions [5]. Among them, the BC of human epidermal growth factor receptor 2− (HER2−) overexpression occurs in about 20–30% of BC and is associated with poor prognoses [6].

With a length of 185 ku transmembrane glycoprotein with tyrosine kinase activities, HER2 is encoded by proto-oncogenes on chromosome 17q21 and consists of 1255 amino acids [7]. Amplification of the HER2 gene is one of the most important factors affecting breast cancer growth and metastasis. After HER2 gene activation, tumor cells can be stimulated by inhibiting apoptosis, promoting their proliferation, increasing their invasiveness, and promoting angiogenesis as well as lymph angiogenesis [8]. Therefore, HER2 is an independent and powerful prognostic indicator for clinical monitoring of breast cancer therapy and is also an important target for tumor-targeted drug selection. Breast cancer patients with HER2 overexpression are characterized by rapid disease progression, short remission period of chemotherapy, poor effects of endocrine therapy, low disease-free survival (DFS), and overall survival (OS) rates [9]. Therefore, recently, targeted therapy for HER2 has been the focus of targeted therapy for breast cancer [10].

Trastuzumab (T), as the gold standard for HER2-positive BC treatment, was the first-line HER2 targeted drug to be approved by the Food and Drug Administration (FDA) and is also the first humanized monoclonal antibody to be approved for HER2-positive BC [11, 12]. Trastuzumab binds HER2’s perimembrane extracellular subdomain IV and exerts antitumor activities through various mechanisms, including inhibiting signal transduction and regulating antibody-dependent cell-mediated cytotoxicity. Moreover, trastuzumab induces internalization and degradation of the HER2 receptor, attracting cytotoxic immune cells into the tumor microenvironment, inhibiting cell growth and proliferation signaling, and ultimately killing tumor cells [13, 14]. In a phase III HERA clinical trial involving 5102 HER2-positive women with early-stage breast cancer, trastuzumab-treated patients exhibited significantly reduced hazard ratios for disease-free survival events (HR=0.76) [15]. Although trastuzumab has changed the paradigm for HER2-positive breast cancer treatment and significantly improved patients’ prognosis, about 35% of patients have natural resistance, and about 70% of patients who initially respond to trastuzumab treatment progress to metastatic disease and develop resistance within 1 year [12, 16]. Moreover, trastuzumab-associated cardiac toxicity limits its clinical applications. Therefore, additional treatments are needed to provide these patients with further clinical benefits.

Lapatinib (L) is a tyrosine kinase inhibitor (TKI) that exerts its anti-tumor effects by competing with intracellular ATP to block the HER2 signal, thereby blocking phosphorylation and downstream changes in molecular pathways [17]. Because of its different mechanisms of action with monoclonal antibodies, it may have some advantages in overcoming drug resistance [18]. In an Alternative III clinical study, patients treated with lapatinib + trastuzumab + aromatase inhibitors (AIs) exhibited significantly longer median progression-free survival (PFS) outcomes than patients treated with trastuzumab + AI (11 months vs. 5.6 months). Moreover, lapatinib + AI-treated patients exhibited longer median PFS than those treated with trastuzumab + AI (8.3 months vs. 5.6 months) [19]. However, in an ALLTO trial [20], the efficacy of lapatinib was inferior to that of trastuzumab. The combination of trastuzumab with lapatinib therapy has also been reported to be more efficacious, relative to trastuzumab therapy. The CHER-Lob and TRIO-US B07 proved that trastuzumab plus lapatinib treatment has a better pathologic complete response (pCR) outcome [21, 22]. However, ALTTO showed that with regard to disease-free survival (DFS), there were no marked differences among trastuzumab plus lapatinib, trastuzumab, and lapatinib therapy groups, with the combination group exhibiting a higher toxicity [20]. There, it has not been conclusively determined whether efficacies of trastuzumab plus lapatinib or lapatinib therapy are not inferior to trastuzumab therapy.

Therefore, we determined whether trastuzumab plus lapatinib or lapatinib therapy is no-inferiority to trastuzumab therapy in HER-positive breast cancer.

## Materials and methods

### Study design

This study was performed in accordance with the Preferred Reporting Items for Systematic Reviews and Meta-Analyses guidelines (PRISMA) [23] and registered in PROSPERO (CRD42021285865).

### Search strategy

Two researchers (YY and LXM) searched relevant studies from PubMed, Embase, the Cochrane library, CNKI, Wan Fang, and Sinomed databases. The Chinese search terms were “ruxianai,” “ruxianzhongliu,” “ruai,” “lapatini,” and “qutuozhudankang.” The English search terms are shown in Table 1.

**Table 1:** PubMed search strategy of lapatinib vs. trastuzumab therapy for HER2-positive breast cancer

Number	Search terms
#1	“neoplasm”[Title/Abstract] OR “carcinoma”[Title/Abstract] OR “cancer”[Title/Abstract] OR “tumor”[Title/Abstract]
#2	“breast”[Title/Abstract]
#3	“lapatinib”[Title/Abstract] OR “Tykerb”[Title/Abstract]
#4	“trastuzumab”[Title/Abstract] OR “Herceptin”[Title/Abstract]
#5	#1 AND #2 AND #3 AND #4

### Inclusion criteria

The inclusion criteria in this study were as follows: (i) patients with HER2-positive (3+ staining with immunohistochemistry or/and fluorescent in situ hybridization (FISH) positive) breast cancer based on clinical, histological, or pathological diagnosis; (ii) treatment of T, L, or T + L arms with chemotherapy combined with trastuzumab, lapatinib, or trastuzumab combined with or followed by lapatinib; (iii) primary outcomes were OS, DFS/event-free survival (EFS), and PFS while secondary outcomes were pCR (ypT0/is ypN0), pCR (ypT0/is ypN0/+), overall response rate (ORR), disease control rate (DCR), rate of breast-conserving surgery (BCS), recurrence-free survival (RFS), cardiac toxicities, and other toxicities; and (iv) randomized controlled trials (RCTs).

### Exclusion criteria

The exclusion criteria were as follows: (i) studies with different chemotherapies among different arms; (ii) conference abstracts and letters among others; and (iii) studies without available outcomes.

### Data extraction and quality assessment

Two researchers extracted the relevant information using a predefined data extraction table, containing literature basic information (trial name, title, author, registration number, publication year), demographic information (number of participants in L + T arm, L arm, and T arm, percentage and number of hormone receptor-positive and hormone receptor-negative participants, tumor stage and diagnosis of patients, inclusion and exclusion criteria), intervention feature information (duration and dose of chemotherapy and anti-HER 2 therapy), and methodological elements (random sequence generation, allocation concealment, blinding of participants and personnel, blinding of outcome assessment, incomplete outcome data, selective reporting, and other bias). The quality of trials was assessed by two researchers using the risk of bias tool of The Cochrane Collaboration [24]. Any disagreements were resolved by discussions with a third researcher.

### Statistical analysis and evidence quality assessment

We used RevMan 5.3 and Stata 14 for all data analyses. A meta-analysis was performed according to the anti-HER2 regimen (L + T versus T or L versus T), respectively. Pooled hazard ratios (HRs) were estimated for survival outcomes, including OS, DFS/EFS, RFS, and PFS while risk ratios (RRs) were determined for dichotomous outcomes, including pCR, ORR, rate of BCS, cardiac toxicities, and other toxicities with 95% confidence intervals (CIs) using the inverse variance or Mantel–Haenszel methods [24]. Heterogeneity was assessed by the *χ*^2^ test and *I*^2^ statistics. A fixed-effects model was used to analyze all effect quantities in this study.

Subgroup analysis was performed using a random-effects model based on the following conditions: tumor stage (I–III or metastatic breast cancer (MBC)), hormone receptor (HR) status, or treatment type (neoadjuvant, adjuvant, or palliative treatment). Sensitivity analysis was performed to identify heterogeneity of main outcomes using the leave-one-out procedure. In addition, a sensitivity analysis was performed to ensure if any of the results were affected by the change of model. After removing obvious heterogeneity studies, a fixed-effects model was used to analyze effect quantities. Publication bias was detected by Egger’s test and considered when *p* ≤ 0.05 [25]. GRADE profiler 3.6 was used to assess the quality of evidence in accordance with five aspects: risk bias, imprecision, indirectness, publication bias, and inconsistency. Evidence qualities were evaluated as high quality, medium quality, low quality, or very low quality.

## Results

### Study selection and characteristics

**Fig. 1:**
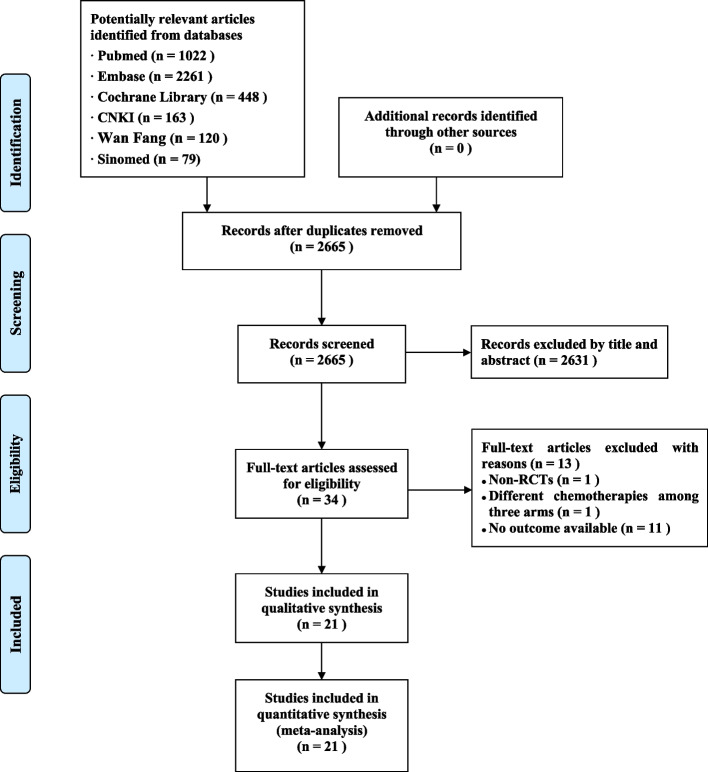
PRISMA diagram of literature searching and screening process

A total of 4093 entries were downloaded from Chinese and English databases. After removing duplicate records and those that were not eligible by reading titles, abstracts, and full-text articles, 21 studies [21–23, 26–43], including 13 RCTs, were identified (Fig. 1).

The included studies had been published between 2012 and 2021. Overall, the studies involved 12,024 eligible participants (T+L: 4817, L: 3570, T: 3637) whose median follow-up time varied from 21.5 months to 9 years. Ten RCTs assessed I–III stage breast cancer [21–23, 26–36, 40–43], while 3 RCTs assessed metastatic breast cancer [37–39]. Nine RCTs, including 7 RCTs with dual HER2 blockade [22, 23, 27–34, 40, 42, 43] and 2 RCTs with single HER2 blockade [35, 36, 41] assessed the role of the anti-HER2 therapy in a neoadjuvant setting [22, 23, 27–36, 40–43], 1 RCT assessed the dual HER2 blockade in an adjuvant setting [21, 26], and 3 RCTs assessed the single HER2 blockade in a palliative setting [37–39]. Characteristics of the included studies are shown in Table 2. More details are shown in Additional file 3: Table S1.

**Table 2:** Main characteristics of the selected studies

Trial	Author	MF	Tumor stage	Chemotherapy (wks)	Anti-HER2 therapy	HER2+ patients	HR+/− patients (%)	Outcomes
ALTTO	Alvaro Moreno-Aspitia 2021 [20] Martine Piccart-Gebhart 2016 [26]	6.9 years	I–III	^*^D1: chemotherapy×(12–18) ^*^D2: A×(9–12) + Taxane×12 ^*^D2B: (Doc + Carb)×18	T+L T→L L T	2093 2091 2100 2097	4805 (57)/3576 (43)	a, c, f, j, k
CHER-Lob	Valentina Guarneri 2021 [21] Valentina Guarneri 2015 [27] Valentina Guarneri 2012 [28]	9 years	II–IIIA	^#^wP×12 + FEC×12	T+L L T	46 39 36	28 (61)/18 (39) 24 (62)/15 (38) 21 (58)/15 (42)	a, d, f, i, j, k
TRIO-US B07	Sara A. Hurvitz 2020 [22]	NR	I–III	^#^Doc×18 + Carb×18	T+L L T	58 36 34	34 (59)/24 (41) 18 (50)/18 (50) 20 (59)/14 (41)	d, j, k
CALGB 40601	Aranzazu Fernandez-Martinez 2020 [29] Lisa A. Carey 2016 [30]	83 months	II–III	^#^wP×16 ^*^AC×(8-12)	T+L L T	118 67 120	70 (59)/48 (41) 39 (58)/28 (42) 70 (58)/50 (42)	a, d, e, f, j, k
NeoALTTO	Jens Huober 2019 [31] Evandro de Azambuja 2014 [32] C. Criscitiello 2013 [33] José Baselga 2012 [34]	6.7 years	I–III	^#^wP×12 ^*^FEC×9	T+L L T	152 154 149	77 (51)/75 (49) 80 (52)/74 (48) 75 (50)/74 (50)	a, c, d, e, g, i, j, k
GeparQuinto	Michael Untch 2018 [35] Michael Untch 2012 [36]	55 months	I–III	^#^EC×12 + Doc×12	L T	308 307	171 (56)/137 (44) 170 (55)/137 (45)	a, c, d, e, f, g, h, i, j, k
WJOG6110B/ELTOP	Toshimi Takano 2018 [37]	44.6 months	MBC	C until progression or intolerable toxicity	L T	43 43	27 (63)/16 (37) 27 (63)/16 (37)	a, b, g, h, j, k
CEREBEL	Xavier Pivot 2015 [38]	NR	MBC	C×NR	L T	271 269	NR NR	a, b, g, h, k
NCIC CTG MA.31	Karen A. Gelmon 2015 [39]	21.5 months	MBC	Taxane×24	L T	270 267	NR NR	a, b, g, h, j, k
EORTC 10054	H. Bonnefoi 2014 [40]	NR	IIA–IIIC	^#^Doc×9 → FEC×9	T+L L T	52 23 53	25 (48)/26 (50) 15 (65)/8 (35) 27 (51)/26 (49)	d, e, g, h, i, j, k
GEICAM/2006-14	E Alba 2014 [41]	NR	I–III	^#^EC×12 → Doc×12	L T	51 48	NR NR	d, e, g, i, j, k
NSABP B-41	André Robidoux 2013 [42]	1.9 years	IIA–IIIA	^#^AC×12 → Doc×16	T+L L T	174 174 181	108 (62)/66 (38) 101 (58)/73 (42) 122 (67)/59 (33)	d, e, g, i, j, k
LPT109096	Frankie Ann Holmes 2013 [43]	NR	II–III	^#^FEC×12 + wP×12	T+L L T	33 34 33	20 (61)/13 (39) 14 (41)/20 (59) 15 (45)/18 (55)	d, g, k

*Adjuvant therapy. ^#^Neoadjuvant therapy. *NR* Not reported, *MBC* Metastatic breast cancer, *MF* Median follow-up, *D* Design, *L* Lapatinib, *T* Trastuzumab, *C* Capecitabine, *A* Anthracycline, *FEC* Fluorouracil + epirubicin + cyclophosphamide, *EC* Epirubicin + cyclophosphamide, *Doc* Docetaxel, *Carb* Carboplatin, *Pal* Paclitaxel, *wP* Weekly paclitaxel, *AC* Doxorubicin + cyclophosphamide, *wk* Week, *HR(+)* hormone receptor positive, *HR(−)* Hormone receptor negative, *a* Overall survival, *b* Progression-free survival, *c* Disease-free survival/event-free survival, *d* pCR (ypT0/is ypN0), *e* pCR (ypT0/is ypN0/+), *f* recurrence-free survival, *g* overall response rate, *h* Disease control rate, *i* rate of breast conserving surgery, *j* cardiac toxicities, *k* other toxicities

### Quality assessment of the included studies

**Fig. 2:**
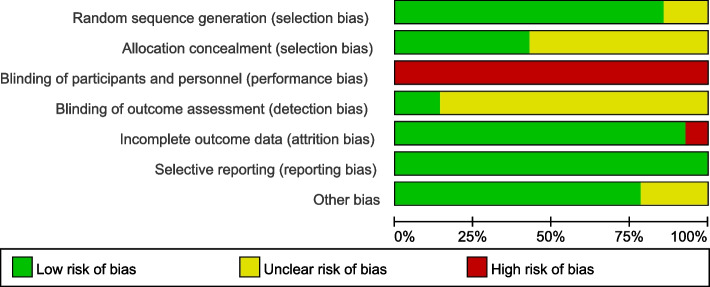
Risk of bias graph

**Fig. 3:**
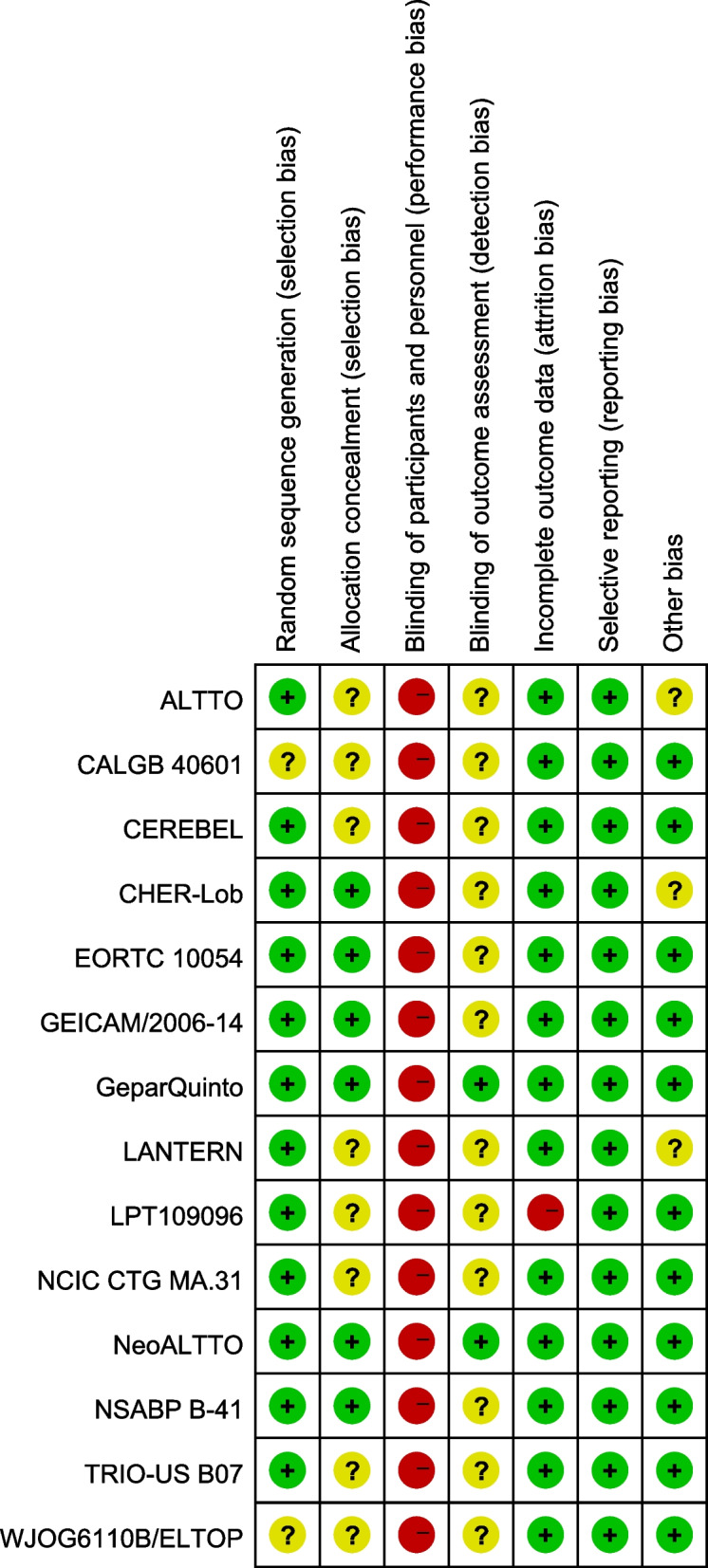
Risk of bias summary

Two RCTs (CALGB 40601 and WJOG6110B/ELTOP) did not describe random sequence generation, while 7 RCTs (ALTTO, CALGB 40601, LPT109096, NCIC CTG MA.31, TRIO-US B07, WJOG6110B/ELTOP, and CEREBEL) did not make detailed illustrations of allocation concealment. Blinding of participants and personnel was not adopted in any of the RCTs. Two RCTs (NeoALTTO and GeparQuinto) adopted blinding of outcome assessment. One RCT (LPT109096) did not report on complete outcome data, while all RCTs were free from reporting bias and 2 RCTs (ALTTO and CHER-Lob) got unclear risk of bias of other bias. Details of risk of bias are shown in Figs. 2 and 3.

### Primary outcomes

#### Overall survival

**Fig. 4:**
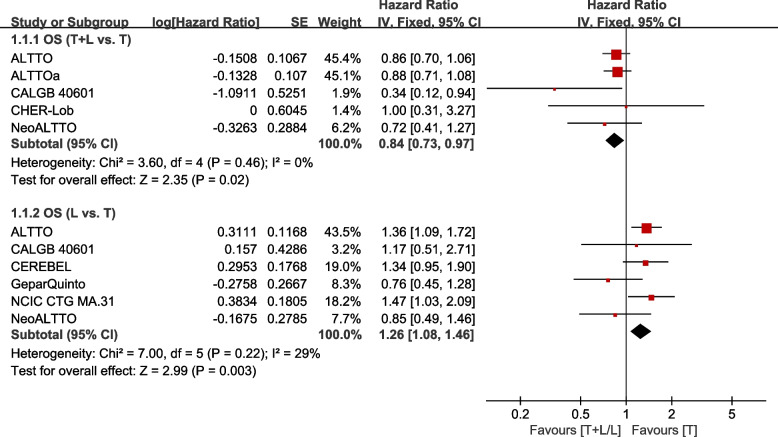
Meta-analysis of OS. ALTTOa: trastuzumab followed by lapatinib group

Eight trials [21, 22, 26, 29, 31, 35, 38, 39] reported data on OS (calculated from randomization to death from any cause or last follow-up) for pooling in meta-analysis. Data from WJOG6110B/ELTOP [37], in which participants were all previously treated with trastuzumab with progression, were excluded. Heterogeneity tests of *p* = 0.46, *I*^2^=0, and *p*=0.22, *I*^2^=29%, were tested in OS (T+L vs. T) and OS (L vs. T). The T+L arm showed significant improvements in OS, compared to the T arm (HR: 0.84, 95% CI: 0.73–0.97, *p*=0.02; Fig. 4). The L arm showed a markedly lower efficacy with regard to OS, compared to T arm (HR: 1.26, 95% CI: 1.08–1.46, *p* =0.003; Fig. 4).

##### Subgroup analysis

**Fig. 5:**
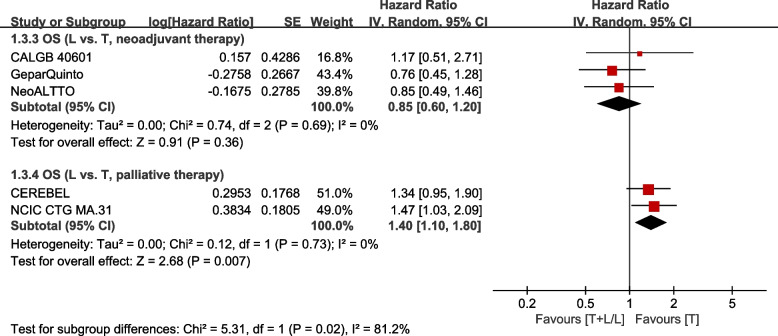
Subgroup analysis of OS in accordance with therapy setting (L vs. T)

The L arm showed no statistical significance in OS of patients with neoadjuvant therapy, compared to T arm (HR: 0.85, 95% CI: 0.60–1.20, *p* =0.36; Fig. 5). The L arm shows no statistical significance in OS of patients with palliative therapy, compared to T arm (HR: 1.40, 95% CI: 1.10–1.80, *p* =0.007; Fig. 5). Subgroup differences were found (interaction test, *p* = 0.02).

#### Progression-free survival

**Fig. 6:**
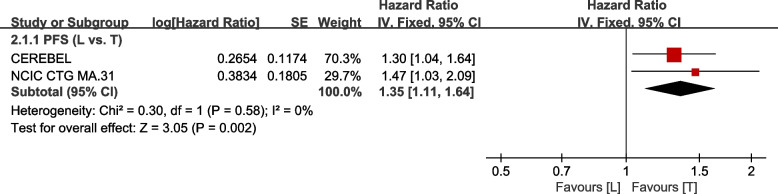
Meta-analysis of PFS

A total of 2 trials [38, 39] provided data on PFS (defined as time from randomization to disease progression) for pooling in the meta-analysis. Data from WJOG6110B/ELTOP [37], in which participants were previously treated with trastuzumab with progression, were excluded. The heterogeneity test of *p*=0.58, *I*^2^=0 in PFS (L vs. T) did not reveal heterogeneity. Compared to the T arm, the L arm showed a lower efficacy with regard to PFS (HR: 1.35, 95% CI: 1.11–1.64, *p*=0.002; Fig. 6).

#### Disease-free survival/event-free survival

**Fig. 7:**
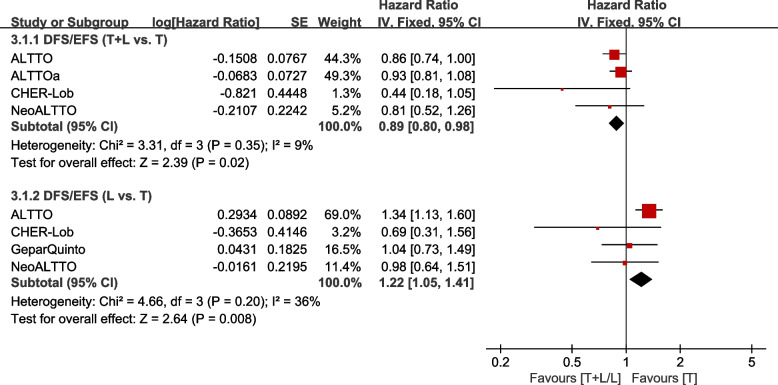
Meta-analysis of DFS/EFS. ALTTOa: trastuzumab followed by lapatinib group

Four trials [21, 26, 31, 35] involving stage I–III patients provided data on DFS/EFS (defined as the time from randomization to recurrence of invasive breast cancer at local, regional, or distant sites; contralateral invasive breast cancer; second non-breast malignancy; or death as a result of any cause, whichever occurred first) for pooling in the meta-analysis. As reported by CHER-Lob [22], RFS was defined as the time from randomization to breast cancer recurrence (loco regional or distant; contralateral BC excluded) or death from any cause, whichever occurred first, which is similar to the definition of DFS/EFS. Therefore, RFS in CHER-Lob [22] was also included for pooling in the meta-analysis. Heterogeneity test of *p*=0.35, *I*^2^=9% in DFS/EFS (T+L vs. T) revealed low heterogeneity. The T+L arm showed significant improvements in DFS/EFS, compared to the T arm (HR: 0.89, 95% CI: 0.80–0.98, *p*=0.02; Fig. 7). Heterogeneity test of *p*=0.20, *I*^2^=36% in DFS/EFS (L vs. T) showed low heterogeneity. Compared to the T arm, the L arm showed a markedly low efficacy with regard to DFS/EFS (HR: 1.22, 95% CI: 1.05–1.41, *p* =0.008; Fig. 7).

### Secondary outcomes

#### pCR (ypT0/is ypN0)

**Fig. 8:**
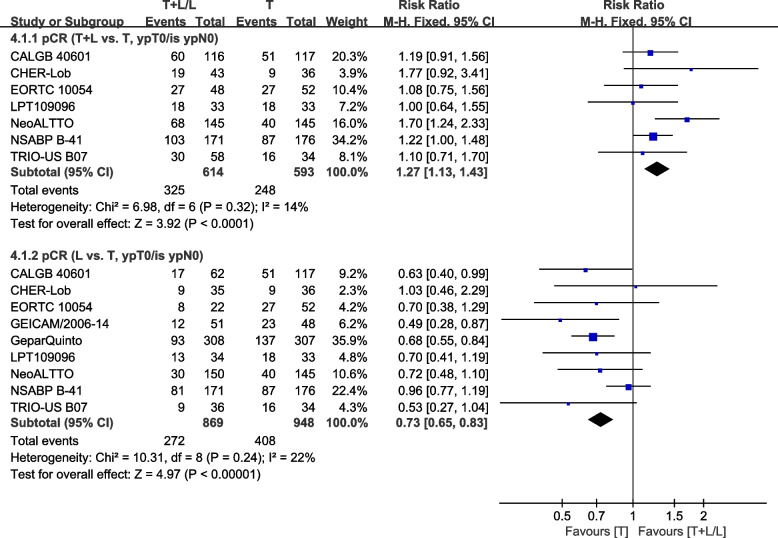
Meta-analysis of pCR(ypT0/is ypN0)

Nine trials [22, 23, 30, 34, 36, 40–43] with neoadjuvant therapy provided data on pCR (ypT0/is ypN0) (defined as the absence of residual invasive tumor in breast and axillary nodes) for pooling in the meta-analysis. Heterogeneity test of *p*=0.32, *I*^2^=14% in pCR (T+L vs. T, ypT0/is ypN0) revealed a low heterogeneity. The T+L arm showed significant improvements in pCR (ypT0/is ypN0), compared to the T arm (RR: 1.27, 95% CI: 1.13–1.43, *p* <0.0001; Fig. 8). Heterogeneity test of *p*=0.24, *I*^2^=22% in pCR (L vs. T, ypT0/is ypN0) showed a low heterogeneity. The T arm showed significant improvements in pCR (ypT0/is ypN0), compared to the L arm (RR: 0.73, 95% CI: 0.65–0.83, *p* <0.00001; Fig. 8).

#### pCR (ypT0/is ypN0/+)

**Fig. 9:**
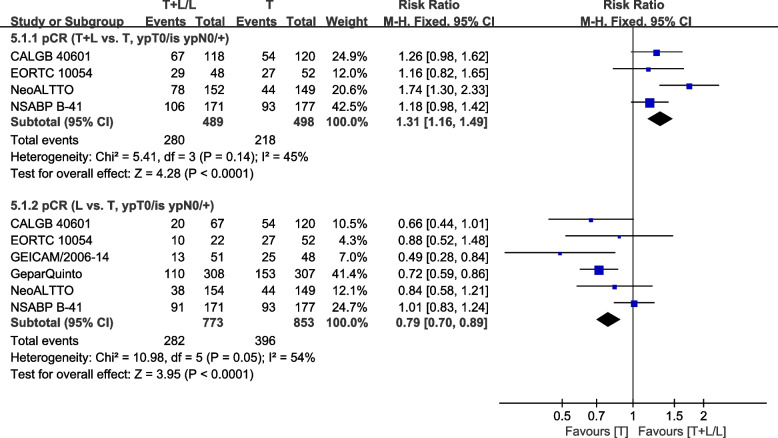
Meta-analysis of pCR(ypT0/is ypN0/+)

Six trials [29, 34, 36, 40–42] with neoadjuvant therapy provided data on pCR (ypT0/is ypN0/+) (defined as the absence of residual invasive tumor in the breast) for pooling in the meta-analysis. Heterogeneity test of *p*=0.14, *I*^2^=45%, in pCR (T+L vs. T, ypT0/is ypN0/+) showed a low heterogeneity. The T+L arm had significant improvements in pCR (ypT0/is ypN0/+), compared to the T arm (RR: 1.31, 95% CI: 1.16–1.49, *p*<0.0001; Fig. 9). Heterogeneity test of *p*=0.05, *I*^2^=54%, in pCR (L vs. T, ypT0/is ypN0/+) showed a high heterogeneity. The T arm showed significant improvements in pCR (ypT0/is ypN0/+), compared to the L arm (RR: 0.79, 95% CI: 0.70–0.89, *p*<0.0001; Fig. 9).

#### Recurrence-free survival

**Fig. 10:**
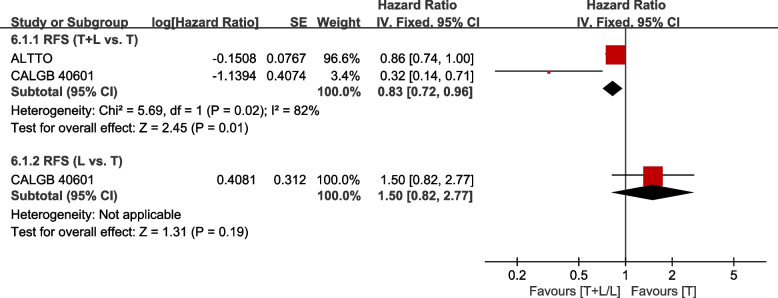
Meta-analysis of RFS

Two trials [21, 29] provided data on RFS (defined as the interval from surgery to ipsilateral invasive breast tumor recurrence, regional recurrence, distant recurrence, or death from any cause, whichever occurred first) for pooling in the meta-analysis. Data from CHER-Lob [22] and GeparQuinto [35] were not pooled in the meta-analysis because RFS was defined as the time from randomization. Heterogeneity test of *p*=0.02, *I*^2^=82%, in RFS (T+L vs. T) showed a high heterogeneity. The T+L arm showed significant improvements in RFS, compared to the T arm (HR: 0.83, 95% CI: 0.72–0.96, *p* =0.01; Fig. 10). CALGB 40601 (30) did not find significant differences between L and T arms with regard to RFS (HR: 1.50, 95% CI: 0.82–2.77, *p* =0.19; Fig. 10).

#### Overall response rate

**Fig. 11:**
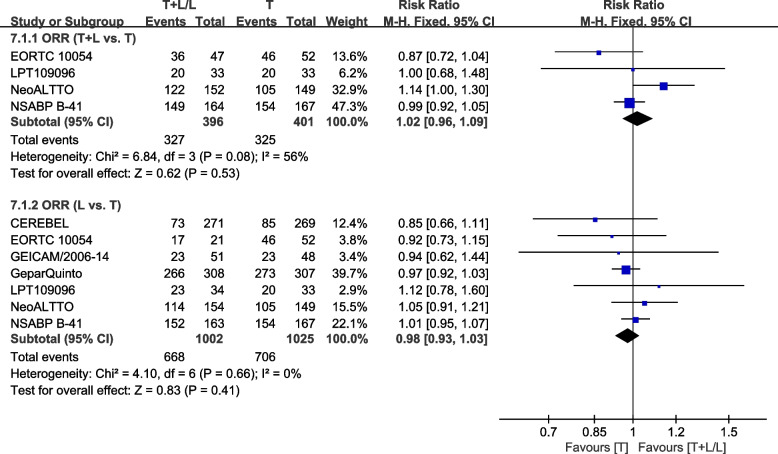
Meta-analysis of ORR

Eight trials [34, 36–38, 40–43] provided data on ORR (based on the World Health Organization (WHO) criteria or the Response Evaluation Criteria in Solid Tumors (RECIST)) for pooling in the meta-analysis. Data from NCIC CTG MA.31 [39] was not pooled in the meta-analysis because HER2-positive and HER2-negative patients were assessed in ORR together. Data from WJOG6110B/ELTOP [37], in which participants were all previously treated with trastuzumab with progression, were excluded. Heterogeneity test of *p*=0.08, *I*^2^=56% in ORR (T+L vs. T) showed a high heterogeneity. Differences in ORR between the T and T+L arms were insignificant (RR: 1.02, 95% CI: 0.96–1.09, *p*=0.53; Fig. 11). Heterogeneity test of *p*=0.66, *I*^2^=0%, in ORR (L vs. T) did not reveal any heterogeneity. Differences in ORR between T and L arms were insignificant (RR: 0.98, 95% CI: 0.93–1.03, *p*=0.41; Fig. 11).

#### Disease control rate

**Fig. 12:**
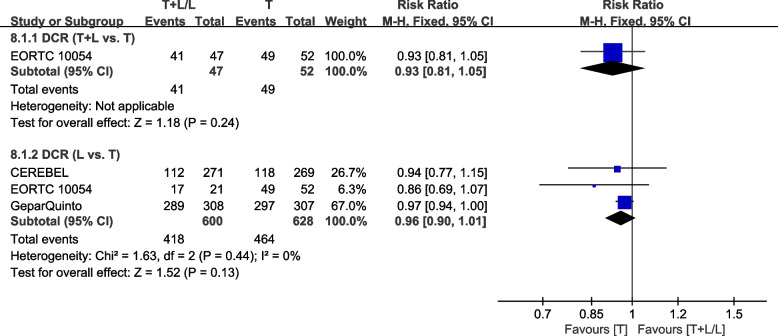
Meta-analysis of DCR

Four trials [35, 37, 38, 40] provided data on DCR (based on the World Health Organization (WHO) criteria or the Response Evaluation Criteria in Solid Tumors (RECIST)) for pooling in the meta-analysis. Data from NCIC CTG MA.31 [39] were not pooled in the meta-analysis because HER2-positive and HER2-negative patients were assessed in DCR together. Data from WJOG6110B/ELTOP [37], in which participants were all previously treated with trastuzumab with progression, were excluded. EORTC 10054 [40] reported that T+L and T arms had comparable DCR rates (Fig. 12). Heterogeneity test of *p*=0.44, *I*^2^=0%, in DCR (L vs. T) did not reveal any heterogeneity. Differences in DCR between the L and T arms were insignificant (RR: 0.96, 95% CI: 0.90–1.01, *p*=0.13; Fig. 12).

#### Rate of breast-conserving surgery

**Fig. 13:**
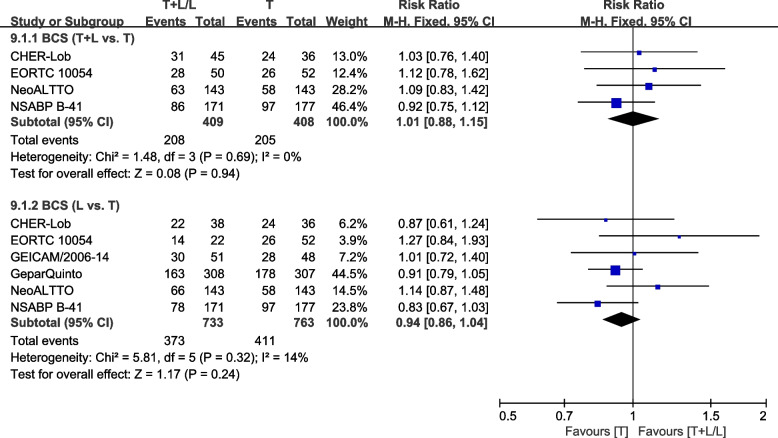
Meta-analysis of rate of BCS

Six trials [27, 33, 36, 40–42] with neoadjuvant therapy reported data on BCS rates for pooling in the meta-analysis. Heterogeneity test of *p*=0.69, *I*^2^=0%, in BCS (T+L vs. T) did not reveal any heterogeneity. Differences in BCS rates between T and T+L arms were not significant (RR: 1.01, 95% CI: 0.88–1.15, *p*=0.94; Fig. 13). Heterogeneity test of *p*=0.32, *I*^2^=14%, in BCS (L vs. T) showed a low heterogeneity. Differences in BCS rates between the L and T arms were insignificant (RR: 0.94, 95% CI: 0.86–1.04, *p*=0.24; Fig. 13).

#### Cardiac toxicities

**Fig. 14:**
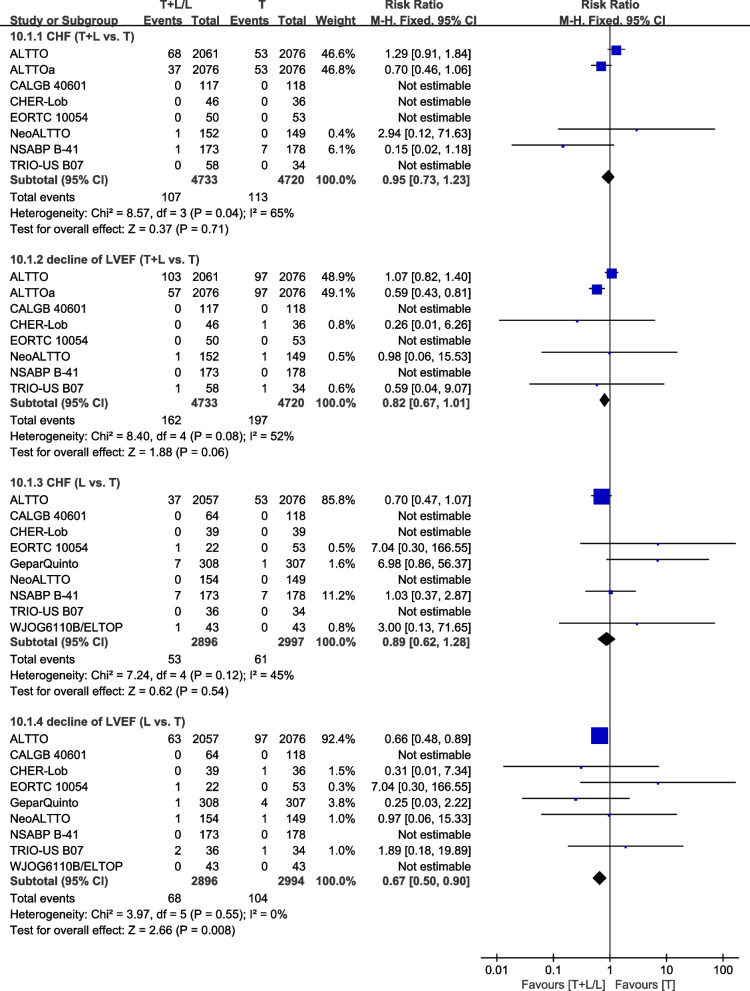
Meta-analysis of cardiac toxicities. ALTTOa: trastuzumab followed by lapatinib group

Nine trials [23, 26, 27, 30, 34, 36, 37, 40, 42] provided data on cardiac toxicities (congestive heart failure (CHF) and decline of left ventricular ejection fraction (LVEF). CHF was defined as Cardiac dysfunction New York Heart Association Class, severe CHF, symptomatic CHF, or confirmed CHF. LVEF decline was defined as reported by the authors of the included studies because different thresholds were used. Data from NCIC CTG MA.31 [39] and GEICAM/2006-14 [41] were not pooled in the meta-analysis because HER2-positive and HER2-negative patients were assessed together. Heterogeneity test of *p*=0.04, *I*^2^=65%, in CHF (T+L vs. T) showed high heterogeneity. Differences in CHF between the T and T+L arms were insignificant (RR: 0.95, 95% CI: 0.73–1.23, *p*=0.71; Fig. 14). Heterogeneity test of *p*=0.08, *I*^2^=52%, in LVEF decline (T+L vs. T) showed high heterogeneity. Differences in LVEF decline between the T+L and T arms were insignificant (RR: 0.82, 95% CI: 0.67–1.01, *p*=0.06; Fig. 14). Heterogeneity test of *p*=0.12, *I*^2^=45%, in CHF (L vs. T) showed a low heterogeneity. Differences in CHF between the L and T arms were insignificant (RR: 0.89, 95% CI: 0.62–1.28, *p*=0.54; Fig. 14). Heterogeneity test of *p*=0.55, *I*^2^=0% in LVEF decline (L vs. T) showed no heterogeneity. Compared to the T arm, the L arm exhibited a lower incidence of LVEF decline (RR: 0.67, 95% CI: 0.50–0.90, *p*=0.008; Fig. 14).

#### Other toxicities

Data on grade III/IV toxicities reported in more than half of the trials were obtained [44]. Eleven trials [23, 26, 28, 30, 32, 36–38, 40, 42, 43] provided data on other toxicities (chemotherapy adverse effects were graded according to the National Cancer Institute Common Terminology Criteria for Adverse Events) for pooling in the meta-analysis. Data from NCIC CTG MA.31 [39] and GEICAM/2006-14 [41] was not pooled in the meta-analysis because HER2-positive and HER2-negative patients were assessed together.

##### Diarrhea

**Fig. 15:**
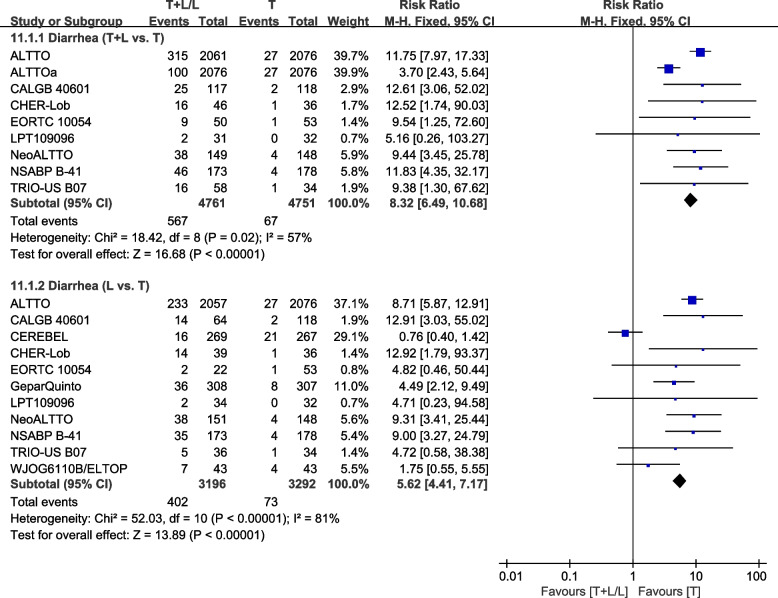
Meta-analysis of diarrhea. ALTTOa: trastuzumab followed by lapatinib group

Eleven trials [23, 26, 28, 30, 32, 36–38, 40, 42, 43] provided data on grade III/IV diarrhea for pooling in the meta-analysis. Heterogeneity test of *p*=0.02, *I*^2^=57%, in diarrhea (T+L vs. T) showed high heterogeneity. Compared to the T arm, the T+L arm showed a higher incidence of grade III/IV diarrhea (RR: 8.32, 95% CI: 6.49–10.68, *p*<0.00001; Fig. 15). Heterogeneity test of *p*<0.00001, *I*^2^=81%, in diarrhea (L vs. T) showed high heterogeneity. The L arm showed a higher incidence of grade III/IV diarrhea, compared to the T arm (RR: 5.62, 95% CI: 4.41–7.17, *p*<0.00001; Fig. 15).

##### Subgroup analysis

**Fig. 16:**
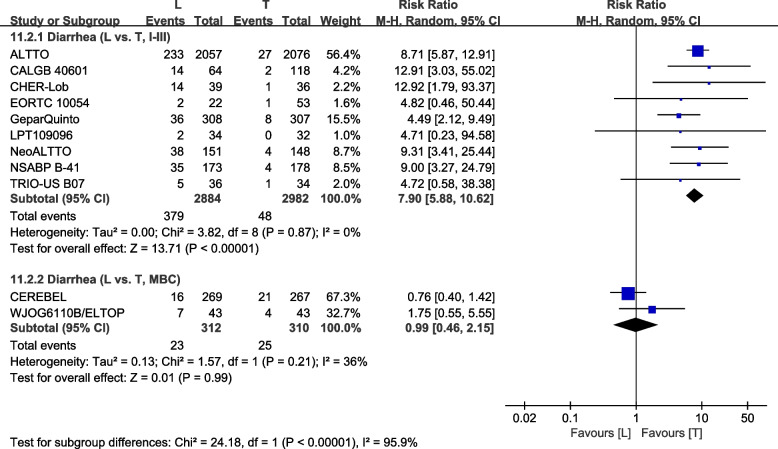
Subgroup analysis of diarrhea in accordance with tumor stage (L vs. T)

Division into subgroups was in accordance with I–III tumor stages [23, 26, 28, 30, 32, 36, 40, 42, 43] or MBC [37, 38]. The L arm had a higher incidence of grade III/IV diarrhea in stage I–III patients (RR: 7.90, 95% CI: 5.88–10.62, *p*<0.00001; Fig. 16). The L arm shows no statistical significance of grade III/IV diarrhea in MBC patients (RR: 0.99, 95% CI: 0.46–2.15, *p*=0.99; Fig. 16). Subgroup differences were found (interaction test, *p*<0.00001).

**Fig. 17:**
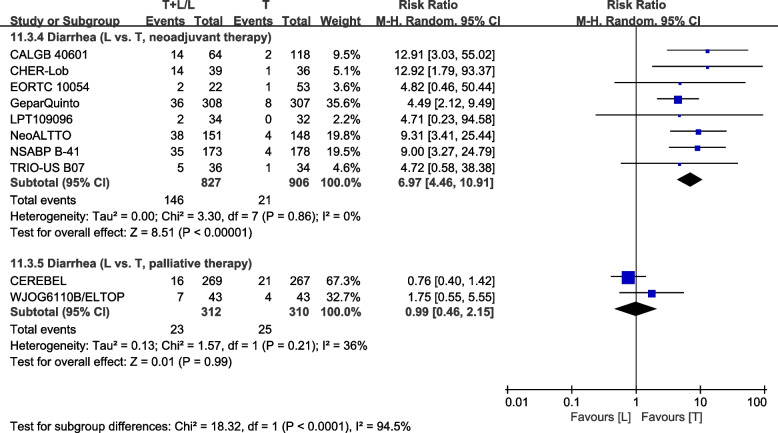
Subgroup analysis of diarrhea in accordance with therapy setting (L vs. T)

Divisions into subgroups were in accordance with treatment type, including neoadjuvant therapy [23, 28, 30, 32, 36, 40, 42, 43] and palliative therapy [37, 38]. The L arm had a higher incidence of grade III/IV diarrhea in patients with neoadjuvant therapy (RR: 6.97, 95% CI: 4.46–10.91 *p*<0.00001; Fig. 17). The L arm shows no statistical significance of grade III/IV diarrhea in patients with palliative therapy (RR: 0.99, 95% CI: 0.46–2.15, *P*=0.99; Fig. 17). Subgroup differences were found (interaction test, *p*<0.00001).

##### Neutropenia

**Fig. 18:**
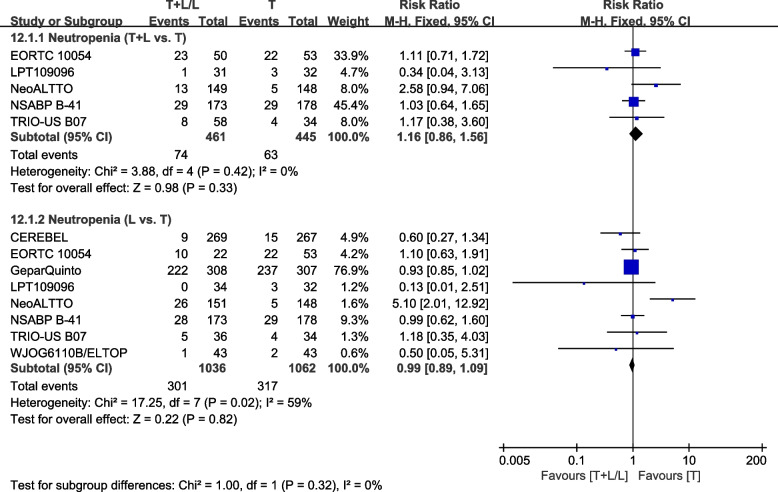
Meta-analysis of neutropenia

Eight trials [23, 32, 36–38, 40, 42, 43] provided data on grade III/IV neutropenia for pooling in the meta-analysis. Heterogeneity test of *p*=0.42, *I*^2^=0%, in neutropenia (T+L vs. T) showed no heterogeneity. Differences in grade III/IV neutropenia between the T and T+L arms were insignificant (RR: 1.16, 95% CI: 0.86–1.56, *p*=0.33; Fig. 18). Heterogeneity test of *p*=0.02, *I*^2^=59%, in neutropenia (L vs. T) showed high heterogeneity. Differences in grade III/IV neutropenia between the T and L arms were insignificant (RR: 0.99, 95% CI: 0.89–1.09, *p*=0.82; Fig. 18).

##### Fatigue

**Fig. 19:**
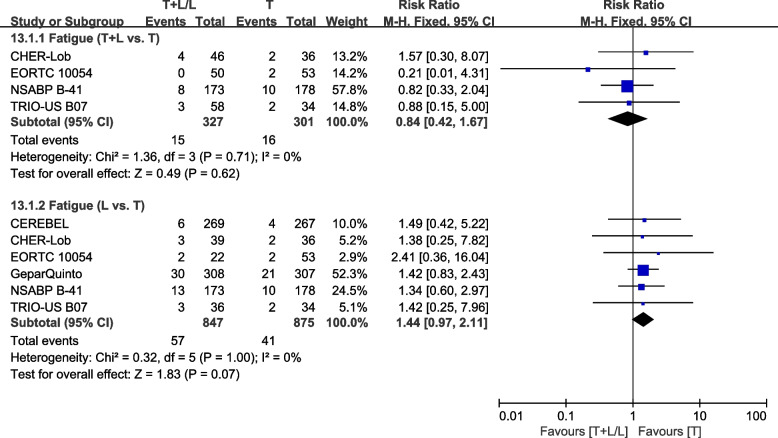
Meta-analysis of fatigue

Six trials [23, 28, 36, 38, 40, 42] provided data on grade III/IV fatigue for pooling in the meta-analysis. Heterogeneity test of *p*=0.71, *I*^2^=0%, in fatigue (T+L vs. T) showed no heterogeneity. Differences in grade III/IV fatigue between the T and T+L arms were insignificant (RR: 0.84, 95% CI: 0.42–1.67, *p*=0.62; Fig. 19). Heterogeneity test of *p*=1.00, *I*^2^=0%, in fatigue (L vs. T) did not reveal any heterogeneity. Differences in grade III/IV fatigue between the T and L arms were insignificant (RR: 1.44, 95% CI: 0.97–2.11, *p*=0.07; Fig. 19).

##### Rash/skin toxicity

**Fig. 20:**
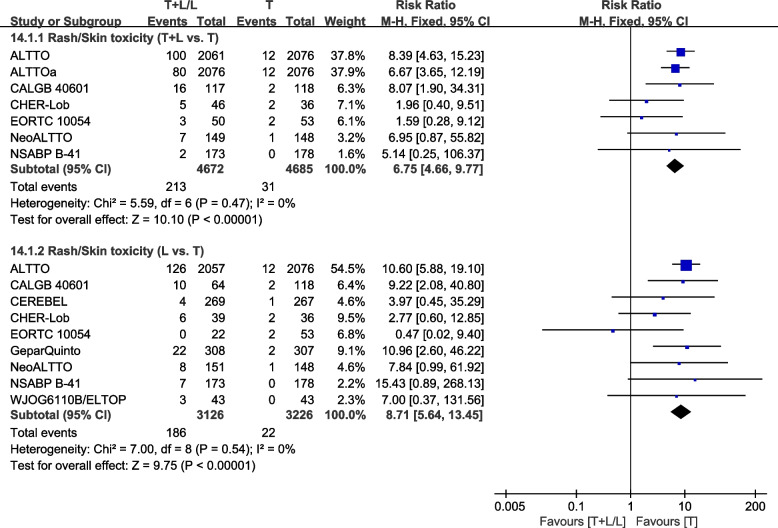
Meta-analysis of rash/skin toxicity

Nine trials [26, 28, 30, 32, 36–38, 40, 42] provided data on grade III/IV rash or skin toxicity for pooling in the meta-analysis. Heterogeneity test of *p*=0.47, *I*^2^=0%, in rash/skin toxicity (T+L vs. T) showed no heterogeneity. The T+L arm had a higher incidence of grade III/IV rash or skin toxicity, when compared to the T arm (RR: 6.75, 95% CI: 4.66–9.77, *p*<0.00001; Fig. 20). Heterogeneity test of *p*=0.54, *I*^2^=0%, in rash/skin toxicity (L vs. T) showed no heterogeneity. The L arm had a higher incidence of grade III/IV rash or skin toxicity, when compared to the T arm (RR: 8.71, 95% CI: 5.64–13.45, *p*<0.00001; Fig. 20).

##### Vomiting

**Fig. 21:**
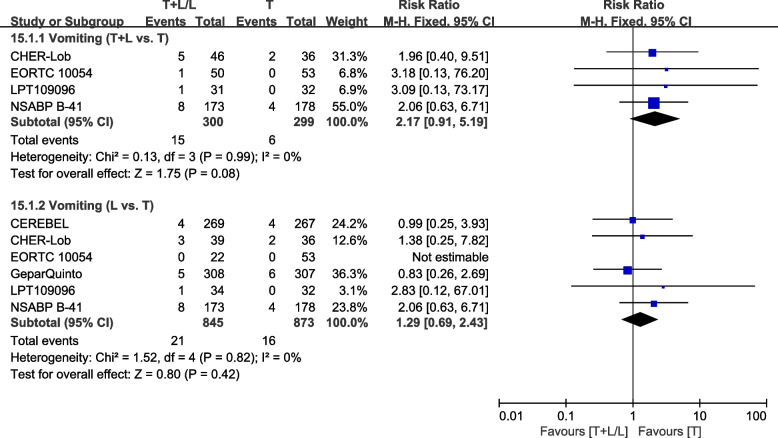
Meta-analysis of vomiting

Six trials [28, 36, 38, 40, 42, 43] provided data on grade III/IV vomiting for pooling in the meta-analysis. Heterogeneity test of *p*=0.99, *I*^2^=0%, in vomiting (T+L vs. T) did not reveal any heterogeneity. Differences in grade III/IV vomiting between the T+L and T arms were not significant (RR: 2.17, 95% CI: 0.91–5.19, *p*=0.08; Fig. 21). Heterogeneity test of *p*=0.82, *I*^2^=0%, in vomiting (L vs. T) did not reveal any heterogeneity. Differences in grade III/IV vomiting between the L and T arms were insignificant (RR: 1.29, 95% CI: 0.69–2.43, *p*=0.42; Fig. 21).

##### Nausea

**Fig. 22:**
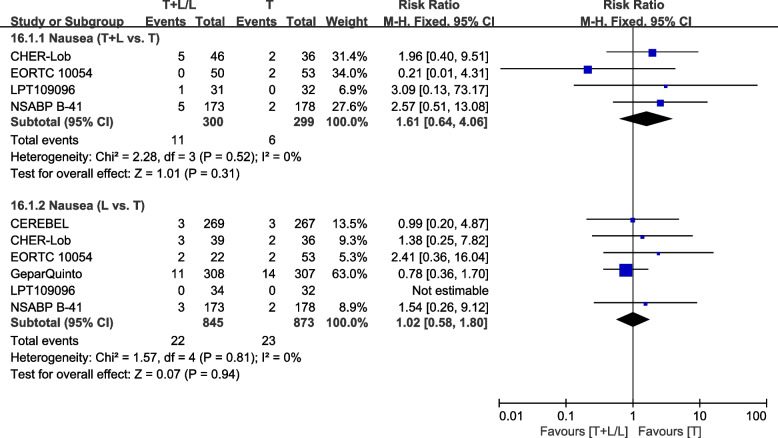
Meta-analysis of nausea

Six trials [28, 36, 38, 40, 42, 43] provided data on grade III/IV nausea for pooling in the meta-analysis. Heterogeneity test of *p*=0.52, *I*^2^=0%, in nausea (T+L vs. T) did not reveal any heterogeneity. Differences in grade III/IV nausea between the T+L and T arms were insignificant (RR: 1.61, 95% CI: 0.64–4.06, *p*=0.31; Fig. 22). Heterogeneity test of *p*=0.81, *I*^2^=0%, in nausea (L vs. T) did not reveal any heterogeneity. Differences in grade III/IV nausea between the T and L arms were insignificant (RR: 1.02, 95% CI: 0.58–1.80, *p*=0.94; Fig. 22).

In this manuscript, we only reported subgroup analysis with a high possibility of subgroup effects. Findings from the meta-analysis and subgroup analyses are summarized in Additional file 4: Table S2. All descriptions and forest plots from subgroup analyses, including from unreported subgroups, are shown in Additional file 1 (Figure S1–S20).

### Publication bias

Publication bias were assessed for the primary outcomes. The Egger’s test did not reveal any publication bias with regard to OS (T+L vs. T) (*t*=−1.43, *p*=0.248, *p*>0.05) and OS (L vs. T) (*t*=−1.71, *p*=0.163, *p*>0.05). Since only 2 trials were included in PFS (L vs. T), the publication bias of this outcome was not determined. The Egger’s test did not reveal any publication bias with regard to DFS/EFS (T+L vs. T; *t*=−2.56, *p*=0.051, *p*>0.05); however, there was a publication bias with regard to DFS/EFS (L vs. T; *t*=−10.88, *p*=0.008, *p*≤0.05). These findings are shown in Additional file 2 (Figure S1–S4).

### Sensitivity analysis

The result of DFS/EFS (L vs. T) revealed a significant difference with the previous result by the change of model [RR = 1.13, 95%CI: 0.91 to 1.42, *p* = 0.27, REM]. The result of RFS (T+L vs. T) revealed a significant difference with the previous result by the change of model [RR = 0.57, 95%CI: 0.22 to 1.48, *p* = 0.25, REM]. The heterogeneity test for pCR (L vs. T, ypT0/is ypN0/+; *p* = 0.05, *I*^2^ = 54%) revealed a high heterogeneity. After excluding data from the NSABP B-41 trial for AC followed by Doc chemotherapy, there was no heterogeneity (*p* = 0.52, *I*^2^ = 0). Therefore, this study is the source of heterogeneity. After deleting the heterogeneity source, the result of pCR (L vs. T, ypT0/is ypN0/+) using the fixed effects model revealed insignificant difference with the previous result [RR = 0.72, 95%CI: 0.62 to 0.83, *p* < 0.00001]. The heterogeneity test for ORR (T+L vs. T) revealed a high heterogeneity (*p* = 0.08, *I*^2^ = 56%). After excluding data from the NeoALTTO trial, which used wP chemotherapy as the neoadjuvant therapy and FEC chemotherapy as the adjuvant therapy, there was no heterogeneity (*p* = 0.41, *I*^2^ = 0). Therefore, this study is the source of heterogeneity. After deleting the source of heterogeneity, the result of ORR (T+L vs. T) using the fixed effects model revealed insignificant difference with the previous result [RR = 0.96, 95%CI: 0.90 to 1.03, *p* = 0.28]. The heterogeneity test for CHF (T+L vs. T) revealed high heterogeneity (*p* = 0.04, *I*^2^ = 65%). After excluding data from the ALTTO trial, which used anti-HER2 therapy as the adjuvant therapy, there was a low heterogeneity (*p* = 0.23, *I*^2^ = 31%). Therefore, this study was the source of heterogeneity. After deleting the source of heterogeneity, the result of CHF (T+L vs. T) using the fixed effects model shows insignificant difference with the previous result [RR = 0.65, 95%CI: 0.44 to 0.97, *p* = 0.04]. The heterogeneity test for LVEF decline (T+L vs. T) revealed a high heterogeneity (*p* = 0.08, *I*^2^ = 52%). After excluding data from the ALTTO trial (ALTTO and ALTTOa), which used anti-HER2 therapy as the adjuvant therapy, there was no heterogeneity (*p* = 0.83, *I*^2^ = 0%). Therefore, this study was the source of heterogeneity. After deleting the source of heterogeneity, the result of LVEF decline (T+L vs. T) using the fixed effects model shows insignificant difference with the previous result [RR = 0.55, 95%CI: 0.11 to 2.73, *p* = 0.46]. The heterogeneity test for diarrhea (T+L vs. T) revealed a high heterogeneity (*p* = 0.02, *I*^2^ = 57%). After excluding data from ALTTOa, which used trastuzumab followed by lapatinib as the anti-HER2 therapy, there was no heterogeneity (*p* = 1.00, *I*^2^ = 0%). Therefore, this study was the heterogeneity source. After deleting the source of heterogeneity, the result of diarrhea (T+L vs. T) using the fixed effects model showed insignificant difference with the previous result [RR = 11.39, 95%CI: 8.30 to 15.63, *p* < 0.00001]. The heterogeneity test for diarrhea (L vs. T) revealed a high heterogeneity (*p* < 0.00001, *I*^2^ = 81%). After excluding data from the CEREBEL trial with a low methodology method, there is little heterogeneity (*p* = 0.34, *I*^2^ = 11%). Therefore, this study was the source of heterogeneity. After deleting the heterogeneity source, the result of diarrhea (L vs. T) using the fixed effects model shows insignificant difference with the previous result [RR = 7.62, 95%CI: 5.73 to 10.11, *p* < 0.00001]. The heterogeneity test for neutropenia (L vs. T) revealed a high heterogeneity (*p* = 0.02, *I*^2^ = 59%). After excluding data from the NeoALTTO trial, which used wP chemotherapy as the neoadjuvant therapy, there was little heterogeneity (*p* = 0.71, *I*^2^ = 0%). Therefore, this study was the source of heterogeneity. After deleting the source of heterogeneity, the result of Neutropenia (L vs. T) using the fixed effects model shows insignificant difference with the previous result [RR = 0.92, 95%CI: 0.83 to 1.02, *p*= 0.10]. All results are stable. Findings from sensitivity analysis are shown in Table 3.

**Table 3:** Sensitivity analysis

Trials	No. of patients	T+L/L	T	RR or HR (95% CI)	*P*-value	*I*^2^ (%)
OS (T+L vs. T)						
ALTTO	4190	2093	2097	0.83 [0.69, 1.01]	0.06	15%
ALTTOa	4188	2091	2097	0.82 [0.68, 0.99]	0.04	12%
CALGB 40601	238	118	120	0.86 [0.75, 0.99]	0.04	0%
CHER-Lob	82	46	36	0.84 [0.73, 0.97]	0.02	15%
NeoALTTO	301	152	149	0.85 [0.74, 0.99]	0.03	9%
Pooled estimate	8999	4500	4499	0.84 [0.73, 0.97]	0.02	0%
OS (L vs. T)						
ALTTO	4197	2100	2097	1.18 [0.97, 1.45]	0.19	35%
CALGB 40601	187	67	120	1.26 [1.08, 1.47]	0.003	43%
CEREBEL	540	271	269	1.24 [1.05, 1.47]	0.01	42%
GeparQuinto	615	308	307	1.32 [1.13, 1.54]	0.0006	0%
NCIC CTG MA.31	537	270	267	1.22 [1.03, 1.44]	0.02	35%
NeoALTTO	303	154	149	1.30 [1.11, 1.52]	0.001	16%
Pooled estimate	6379	3170	3209	1.26 [1.08, 1.46]	0.003	29%
PFS (L vs. T)						
CEREBEL	540	271	269	1.47 [1.03, 2.09]	0.03	/
NCIC CTG MA.31	537	270	267	1.30 [1.04, 1.64]	0.02	/
Pooled estimate	1077	541	536	1.35 [1.11, 1.64]	0.002	0%
DFS/EFS (T+L vs. T)						
ALTTO	4190	2093	2097	0.91 [0.79, 1.04]	0.15	35%
ALTTOa	4188	2091	2097	0.84 [0.73, 0.97]	0.01	10%
CHER-Lob	82	46	36	0.89 [0.81, 0.99]	0.03	0%
NeoALTTO	301	152	149	0.89 [0.80, 0.99]	0.03	36%
Pooled estimate	8761	4382	4379	0.89 [0.80, 0.98]	0.02	9%
DFS/EFS (L vs. T) (REM)						
ALTTO	4197	2100	2097	0.98 [0.75, 1.27]	0.88	0%
CHER-Lob	75	39	36	1.20 [0.98, 1.46]	0.08	28%
GeparQuinto	615	308	307	1.12 [0.81, 1.55]	0.49	48%
NeoALTTO	303	154	149	1.15 [0.87, 1.53]	0.31	45%
Pooled estimate	5190	2601	2589	1.13 [0.91, 1.42]	0.27	36%
pCR (T+L vs. T, ypT0/is ypN0)						
CALGB 40601	233	116	117	1.29 [1.13, 1.48]	0.0002	27%
CHER-Lob	79	43	36	1.25 [1.11, 1.41]	0.0003	14%
EORTC 10054	100	48	52	1.29 [1.14, 1.47]	< 0.0001	21%
LPT109096	66	33	33	1.29 [1.14, 1.46]	< 0.0001	16%
NeoALTTO	290	145	145	1.19 [1.05, 1.35]	0.008	0%
NSABP B-41	347	171	176	1.30 [1.12, 1.51]	0.0007	28%
TRIO-US B07	92	58	34	1.29 [1.14, 1.46]	< 0.0001	25%
Pooled estimate	1207	614	593	1.27 [1.13, 1.43]	< 0.0001	14%
pCR (L vs. T, ypT0/is ypN0)						
CALGB 40601	179	62	117	0.74 [0.65, 0.84]	<0.00001	28%
CHER-Lob	71	35	36	0.73 [0.64, 0.82]	<0.00001	28%
EORTC 10054	74	22	52	0.73 [0.65, 0.83]	<0.00001	32%
GEICAM/2006-14	99	51	48	0.75 [0.66, 0.85]	<0.00001	14%
GeparQuinto	615	308	307	0.76 [0.66, 0.89]	0.0005	23%
LPT109096	67	34	33	0.73 [0.65, 0.83]	<0.00001	32%
NeoALTTO	295	150	145	0.73 [0.65, 0.83]	<0.00001	32%
NSABP B-41	347	171	176	0.67 [0.58, 0.77]	<0.00001	0%
TRIO-US B07	70	36	34	0.74 [0.66, 0.84]	<0.00001	24%
Pooled estimate	1817	869	948	0.73 [0.65, 0.83]	<0.00001	22%
pCR (T+L vs. T, ypT0/is ypN0/+)						
CALGB 40601	238	118	120	1.33 [1.15, 1.54]	<0.0001	63%
EORTC 10054	100	48	52	1.33 [1.17, 1.52]	<0.0001	61%
NeoALTTO	301	152	149	1.20 [1.05, 1.38]	0.008	0%
NSABP B-41	348	171	177	1.41 [1.19, 1.67]	<0.0001	49%
Pooled estimate	987	489	498	1.31 [1.16, 1.49]	<0.0001	45%
pCR (L vs. T, ypT0/is ypN0/+)						
CALGB 40601	187	67	120	0.80 [0.71, 0.91]	0.0005	60%
EORTC 10054	74	22	52	0.79 [0.70, 0.89]	<0.0001	63%
GEICAM/2006-14	99	51	48	0.81 [0.72, 0.92]	0.0007	47%
GeparQuinto	615	308	307	0.84 [0.72, 0.98]	0.02	53%
NeoALTTO	303	154	149	0.78 [0.69, 0.89]	0.0001	64%
NSABP B-41	348	171	177	0.72 [0.62, 0.83]	<0.00001	0%
Pooled estimate	1626	773	853	0.79 [0.70, 0.89]	<0.0001	54%
RFS (T+L vs. T) (REM)						
ALTTO	4190	2093	2097	0.32 [0.14, 0.71]	0.005	/
CALGB 40601	238	118	120	0.86 [0.74, 1.00]	0.05	/
Pooled estimate	4428	2211	2217	0.57 [0.22, 1.48]	0.25	82%
ORR (T+L vs. T)						
EORTC 10054	99	47	52	1.04 [0.98, 1.12]	0.21	58%
LPT109096	66	33	33	1.02 [0.96, 1.09]	0.50	71%
NeoALTTO	301	152	149	0.96 [0.90, 1.03]	0.28	0%
NSABP B-41	331	164	167	1.05 [0.95, 1.17]	0.34	65%
Pooled estimate	797	396	401	1.02 [0.96, 1.09]	0.53	56%
ORR (L vs. T)						
CEREBEL	540	271	269	1.00 [0.95, 1.04]	0.88	0%
EORTC 10054	73	21	52	0.98 [0.93, 1.03]	0.48	0%
GEICAM/2006-14	99	51	48	0.98 [0.93, 1.03]	0.43	0%
GeparQuinto	615	308	307	0.98 [0.91, 1.06]	0.66	0%
LPT109096	67	34	33	0.97 [0.93, 1.03]	0.32	0%
NeoALTTO	303	154	149	0.97 [0.91, 1.02]	0.20	0%
NSABP B-41	330	163	167	0.97 [0.91, 1.03]	0.34	0%
Pooled estimate	2027	1002	1025	0.98 [0.93, 1.03]	0.41	0%
DCR (L vs. T)						
CEREBEL	540	271	269	0.96 [0.93, 1.00]	0.03	24%
EORTC 10054	73	21	52	0.96 [0.91, 1.02]	0.21	0%
GeparQuinto	615	308	307	0.93 [0.79, 1.09]	0.36	0%
Pooled estimate	1228	600	628	0.96 [0.90, 1.01]	0.13	0%
Rate of BCS (T+L vs. T)						
CHER-Lob	81	45	36	1.00 [0.86, 1.16]	0.99	0%
EORTC 10054	102	50	52	0.99 [0.86, 1.14]	0.88	0%
NeoALTTO	286	143	143	0.97 [0.83, 1.14]	0.73	0%
NSABP B-41	348	171	177	1.08 [0.90, 1.30]	0.40	0%
Pooled estimate	817	409	408	1.01 [0.88, 1.15]	0.94	0%
Rate of BCS (L vs. T)						
CHER-Lob	74	38	36	0.95 [0.86, 1.05]	0.31	28%
EORTC 10054	74	22	52	0.93 [0.84, 1.03]	0.16	0%
GEICAM/2006-14	99	51	48	0.94 [0.85, 1.04]	0.22	29%
GeparQuinto	615	308	307	0.97 [0.85, 1.11]	0.64	27%
NeoALTTO	286	143	143	0.91 [0.82, 1.01]	0.08	0%
NSABP B-41	348	171	177	0.98 [0.88, 1.09]	0.70	4%
Pooled estimate	1496	733	763	0.94 [0.86, 1.04]	0.24	14%
CHF (T+L vs. T)						
TRIO-US B07	92	58	34	0.95 [0.73, 1.23]	0.71	65%
NSABP B-41	351	173	178	1.00 [0.77, 1.31]	0.98	62%
NeoALTTO	301	152	149	0.94 [0.73, 1.22]	0.66	75%
EORTC 10054	103	50	53	0.95 [0.73, 1.23]	0.71	65%
CHER-Lob	82	46	36	0.95 [0.73, 1.23]	0.71	65%
CALGB 40601	235	117	118	0.95 [0.73, 1.23]	0.71	65%
ALTTOa	4152	2076	2076	1.17 [0.84, 1.65]	0.35	55%
ALTTO	4137	2061	2076	0.65 [0.44, 0.97]	0.04	31%
Pooled estimate	9453	4733	4720	0.95 [0.73, 1.23]	0.71	65%
CHF (L vs. T)						
WJOG6110B/ELTOP	86	43	43	0.88 [0.61, 1.26]	0.47	55%
TRIO-US B07	70	36	34	0.89 [0.62, 1.28]	0.54	45%
NSABP B-41	351	173	178	0.88 [0.60, 1.28]	0.50	58%
NeoALTTO	303	154	149	0.89 [0.62, 1.28]	0.54	45%
GeparQuinto	615	308	307	0.79 [0.55, 1.15]	0.22	2%
EORTC 10054	75	22	53	0.86 [0.60, 1.24]	0.43	45%
CHER-Lob	78	39	39	0.89 [0.62, 1.28]	0.54	45%
CALGB 40601	182	64	118	0.89 [0.62, 1.28]	0.54	45%
ALTTO	4133	2057	2076	2.03 [0.92, 4.48]	0.08	18%
Pooled estimate	5893	2896	2997	0.89 [0.62, 1.28]	0.54	45%
Decline of LVEF (T+L vs. T)						
TRIO-US B07	92	58	34	0.82 [0.67, 1.01]	0.06	64%
NSABP B-41	351	173	178	0.82 [0.67, 1.01]	0.08	52%
NeoALTTO	301	152	149	0.82 [0.67, 1.01]	0.06	64%
EORTC 10054	103	50	53	0.82 [0.67, 1.01]	0.06	52%
CHER-Lob	82	46	36	0.83 [0.67, 1.01]	0.07	62%
CALGB 40601	235	117	118	0.82 [0.67, 1.01]	0.06	52%
ALTTOa	4152	2076	2076	1.05 [0.80, 1.37]	0.72	0%
ALTTO	4137	2061	2076	0.59 [0.43, 0.80]	0.0009	0%
Pooled estimate	9453	4733	4720	0.82 [0.67, 1.01]	0.06	52%
Decline of LVEF (L vs. T)						
WJOG6110B/ELTOP	86	43	43	0.67 [0.50, 0.90]	0.008	0%
TRIO-US B07	70	36	34	0.66 [0.49, 0.89]	0.006	0%
NSABP B-41	351	173	178	0.67 [0.50, 0.90]	0.008	0%
NeoALTTO	303	154	149	0.67 [0.49, 0.90]	0.008	0%
GeparQuinto	615	308	307	0.68 [0.51, 0.93]	0.01	0%
EORTC 10054	75	22	53	0.65 [0.48, 0.88]	0.005	0%
CHER-Lob	75	39	36	0.67 [0.50, 0.91]	0.010	0%
CALGB 40601	182	64	118	0.67 [0.50, 0.90]	0.008	0%
ALTTO	4133	2057	2076	0.82 [0.30, 2.24]	0.70	0%
Pooled estimate	5890	2896	2994	0.67 [0.50, 0.90]	0.008	0%
Diarrhea (T+L vs. T)						
ALTTO	4137	2061	2076	6.06 [4.37, 8.41]	<0.00001	28%
ALTTOa	4152	2076	2076	11.39 [8.30, 15.63]	<0.00001	0%
CALGB 40601	235	117	118	8.19 [6.36, 10.55]	<0.00001	61%
CHER-Lob	82	46	36	8.25 [6.42, 10.61]	<0.00001	61%
EORTC 10054	103	50	53	8.31 [6.46, 10.67]	<0.00001	62%
LPT109096	63	31	32	8.35 [6.50, 10.72]	<0.00001	62%
NeoALTTO	297	149	148	8.25 [6.38, 10.67]	<0.00001	62%
NSABP B-41	351	173	178	8.11 [6.27, 10.49]	<0.00001	60%
TRIO-US B07	92	58	34	8.30 [6.46, 10.67]	<0.00001	62%
Pooled estimate	9512	4761	4751	8.32 [6.49, 10.68]	<0.00001	57%
Diarrhea (L vs. T)						
ALTTO	4133	2057	2076	3.81 [2.78, 5.21]	<0.00001	76%
CALGB 40601	182	64	118	5.48 [4.28, 7.02]	<0.00001	82%
CEREBEL	536	269	267	7.62 [5.73, 10.11]	<0.00001	11%
CHER-Lob	75	39	36	5.52 [4.32, 7.05]	<0.00001	82%
EORTC 10054	75	22	53	5.63 [4.41, 7.19]	<0.00001	83%
GeparQuinto	615	308	307	5.76 [4.45, 7.46]	<0.00001	83%
LPT109096	66	34	32	5.63 [4.41, 7.19]	<0.00001	83%
NeoALTTO	299	151	148	5.40 [4.20, 6.95]	<0.00001	82%
NSABP B-41	351	173	178	5.43 [4.22, 6.98]	<0.00001	82%
TRIO-US B07	70	36	34	5.64 [4.41, 7.20]	<0.00001	83%
WJOG6110B/ELTOP	86	43	43	5.85 [4.55, 7.51]	<0.00001	81%
Pooled estimate	6488	3196	3292	5.62 [4.41, 7.17]	<0.00001	81%
Neutropenia (T+L vs. T)						
EORTC 10054	103	50	53	1.19 [0.81, 1.74]	0.39	22%
LPT109096	63	31	32	1.20 [0.89, 1.62]	0.23	0%
NeoALTTO	297	149	148	1.04 [0.76, 1.41]	0.82	0%
NSABP B-41	351	173	178	1.27 [0.87, 1.85]	0.22	18%
TRIO-US B07	92	58	34	1.16 [0.85, 1.57]	0.35	23%
Pooled estimate	906	461	445	1.16 [0.86, 1.56]	0.33	0%
Neutropenia (L vs. T)						
CEREBEL	536	269	267	1.01 [0.91, 1.11]	0.86	64%
EORTC 10054	75	22	53	0.98 [0.89, 1.09]	0.76	65%
GeparQuinto	615	308	307	1.17 [0.88, 1.56]	0.28	61%
LPT109096	66	34	32	1.00 [0.90, 1.10]	0.98	62%
NeoALTTO	299	151	148	0.92 [0.83, 1.02]	0.10	0%
NSABP B-41	351	173	178	0.99 [0.89, 1.09]	0.82	65%
TRIO-US B07	70	36	34	0.99 [0.89, 1.09]	0.78	65%
WJOG6110B/ELTOP	86	43	43	0.99 [0.90, 1.10]	0.87	65%
Pooled estimate	2098	1036	1062	0.99 [0.89, 1.09]	0.82	59%
Fatigue (T+L vs. T)						
CHER-Lob	82	46	36	0.73 [0.34, 1.57]	0.42	0%
EORTC 10054	103	50	53	0.95 [0.46, 1.94]	0.88	0%
NSABP B-41	351	173	178	0.87 [0.30, 2.48]	0.79	0%
TRIO-US B07	92	58	34	0.84 [0.40, 1.76]	0.64	0%
Pooled estimate	628	327	301	0.84 [0.42, 1.67]	0.62	0%
Fatigue (L vs. T)						
CEREBEL	536	269	267	1.43 [0.95, 2.15]	0.09	0%
CHER-Lob	75	39	36	1.44 [0.97, 2.14]	0.07	0%
EORTC 10054	75	22	53	1.41 [0.95, 2.09]	0.09	0%
GeparQuinto	615	308	307	1.45 [0.83, 2.54]	0.20	0%
NSABP B-41	351	173	178	1.47 [0.94, 2.29]	0.09	0%
TRIO-US B07	70	36	34	1.44 [0.97, 2.14]	0.07	0%
Pooled estimate	1722	847	875	1.44 [0.97, 2.11]	0.07	0%
Rash/Skin toxicity (T+L vs. T)						
ALTTO	4137	2061	2076	5.75 [3.57, 9.24]	<0.00001	0%
ALTTOa	4152	2076	2076	6.80 [4.25, 10.87]	<0.00001	11%
CALGB 40601	235	117	118	6.66 [4.54, 9.77]	<0.00001	9%
CHER-Lob	82	46	36	7.11 [4.85, 10.44]	<0.00001	0%
EORTC 10054	103	50	53	7.08 [4.84, 10.38]	<0.00001	0%
NeoALTTO	297	149	148	6.74 [4.63, 9.82]	<0.00001	11%
NSABP B-41	351	173	178	6.77 [4.66, 9.84]	<0.00001	10%
Pooled estimate	9357	4672	4685	6.75 [4.66, 9.77]	<0.00001	0%
Rash/Skin toxicity (L vs. T)						
ALTTO	4133	2057	2076	6.45 [3.38, 12.29]	<0.00001	0%
CALGB 40601	182	64	118	8.67 [5.51, 13.65]	<0.00001	0%
CEREBEL	536	269	267	8.93 [5.73, 13.93]	<0.00001	0%
CHER-Lob	75	39	36	9.33 [5.91, 14.72]	<0.00001	0%
EORTC 10054	75	22	53	9.31 [5.94, 14.58]	<0.00001	0%
GeparQuinto	615	308	307	8.48 [5.37, 13.38]	<0.00001	0%
NeoALTTO	299	151	148	8.75 [5.61, 13.65]	<0.00001	0%
NSABP B-41	351	173	178	8.55 [5.51, 13.28]	<0.00001	0%
WJOG6110B/ELTOP	86	43	43	8.75 [5.63, 13.58]	<0.00001	0%
Pooled estimate	6352	3126	3226	8.71 [5.64, 13.45]	<0.00001	0%
Vomiting (T+L vs. T)						
CHER-Lob	82	46	36	2.27 [0.80, 6.44]	0.12	0%
EORTC 10054	103	50	53	2.10 [0.85, 5.19]	0.11	0%
LPT109096	63	31	32	2.11 [0.85, 5.21]	0.11	0%
NSABP B-41	351	173	178	2.31 [0.64, 8.37]	0.20	0%
Pooled estimate	599	300	299	2.17 [0.91, 5.19]	0.08	0%
Vomiting (L vs. T)						
CEREBEL	536	269	267	1.39 [0.68, 2.84]	0.37	0%
CHER-Lob	75	39	36	1.28 [0.65, 2.53]	0.48	0%
EORTC 10054	75	22	53	1.29 [0.69, 2.43]	0.42	0%
GeparQuinto	615	308	307	1.56 [0.73, 3.33]	0.25	0%
LPT109096	66	34	32	1.24 [0.65, 2.38]	0.51	0%
NSABP B-41	351	173	178	1.05 [0.49, 2.26]	0.89	0%
Pooled estimate	1718	845	873	1.29 [0.69, 2.43]	0.42	0%
Nausea (T+L vs. T)						
CHER-Lob	82	46	36	1.45 [0.46, 4.55]	0.52	12%
EORTC 10054	103	50	53	2.33 [0.80, 6.76]	0.12	0%
LPT109096	63	31	32	1.50 [0.57, 3.96]	0.41	7%
NSABP B-41	351	173	178	1.24 [0.40, 3.91]	0.71	0%
Pooled estimate	599	300	299	1.61 [0.64, 4.06]	0.31	0%
Nausea (L vs. T)						
CEREBEL	536	269	267	1.03 [0.56, 1.89]	0.94	0%
CHER-Lob	75	39	36	0.98 [0.54, 1.80]	0.96	0%
EORTC 10054	75	22	53	0.94 [0.52, 1.72]	0.85	0%
GeparQuinto	615	308	307	1.43 [0.60, 3.37]	0.42	0%
LPT109096	66	34	32	1.02 [0.58, 1.80]	0.94	0%
NSABP B-41	351	173	178	0.97 [0.53, 1.77]	0.92	0%
Pooled estimate	1718	845	873	1.02 [0.58, 1.80]	0.94	0%

### Evidence quality assessment

Thirty-seven outcomes were assessed by GRADE. Risk bias: Almost all outcomes were considered serious risk due to unclear allocation concealment of the included studies, except outcomes of BCS rates for T+L vs. T and L vs. T. Inconsistency: High heterogeneities were found in outcomes of pCR (L vs. T, ypT0/is ypN0/+), RFS (T+L vs. T), ORR (T+L vs. T), CHF (T+L vs. T), LVEF decline (T+L vs. T), diarrhea (T+L vs. T), diarrhea (L vs. T), and neutropenia (L vs. T). Thus, these outcomes were considered serious risk of inconsistency. Indirectness: All outcomes had no significant indirectness, because all trials were direct comparisons. Imprecision: Outcomes of OS (L vs. T, neoadjuvant therapy), DFS/EFS (L vs. T), RFS (T+L vs. T), ORR (T+L vs. T), ORR (L vs. T), DCR (L vs. T), rate of BCS (T+L vs. T), rate of BCS (L vs. T), CHF (T+L vs. T), CHF (L vs. T), LVEF decline (T+L vs. T), diarrhea (T+L vs. MBC), diarrhea (T+L vs. palliative therapy), neutropenia (T+L vs. T), neutropenia (L vs. T), fatigue (T+L vs. T), fatigue (L vs. T), vomiting (T+L vs. T), vomiting (L vs. T), nausea (T+L vs. T), and nausea (L vs. T) were considered serious risk of imprecision due to insufficient sample size. Publication bias: DFS/EFS (L vs. T), CHF (L vs. T) and nausea (L vs. T) exhibited a publication bias. Overall: No outcomes had high-quality evidence, 15 outcomes had moderate-quality evidence, 14 outcomes had low-quality evidence, and 8 outcomes had very low-quality evidence (Table 4).

**Table 4:** GRADE evidence profile of outcomes

Outcome	Number of studies	Assessment of evidence quality					Number of participants	Effect(95%CI)	Evidence quality
		Risk bias	Inconsistency	Indirectness	Imprecision	Publication bias			
OS (T+L vs. T)	4	Serious	No	No	No	Undetected	8999	HR = 0.84 [0.73, 0.97]	Moderate
OS (L vs. T)	6	Serious	No	No	No	Undetected	6379	HR = 1.26 [1.08, 1.46]	Moderate
OS (L vs. T, neoadjuvant therapy)	3	Serious	No	No	Serious	Undetected	1105	HR = 0.85 [0.60, 1.20]	Low
OS (L vs. T, palliative therapy)	2	Serious	No	No	No	Undetected	1077	HR = 1.40 [1.10, 1.80]	Moderate
PFS (L vs. T)	2	Serious	No	No	No	Undetected	1077	HR = 1.35 [1.11, 1.64]	Moderate
DFS/EFS (T+L vs. T)	3	Serious	No	No	No	Undetected	8761	HR = 0.89 [0.80, 0.98]	Moderate
DFS/EFS (L vs. T)	4	Serious	No	No	Serious	Detected	5190	HR = 1.22 [1.05, 1.41]	Very low
pCR (T+L vs. T, ypT0/is ypN0)	7	Serious	No	No	No	Undetected	1207	RR = 1.27 [1.13, 1.43]	Moderate
pCR (L vs. T, ypT0/is ypN0)	9	Serious	No	No	No	Undetected	1817	RR = 0.73 [0.65, 0.83]	Moderate
pCR (T+L vs. T, ypT0/is ypN0/+)	4	Serious	No	No	No	Undetected	987	RR = 1.31 [1.16, 1.49]	Moderate
pCR (L vs. T, ypT0/is ypN0/+)	6	Serious	Serious	No	No	Undetected	1626	RR = 0.79 [0.70, 0.89]	Low
RFS (T+L vs. T)	2	Serious	Serious	No	Serious	Undetected	4428	HR = 0.83 [0.72, 0.96]	Very low
ORR (T+L vs. T)	4	Serious	Serious	No	Serious	Undetected	797	RR = 1.02 [0.96, 1.09]	Very low
ORR (L vs. T)	7	Serious	No	No	Serious	Undetected	2027	RR = 0.98 [0.93, 1.03]	Low
DCR (L vs. T)	3	Serious	No	No	Serious	Undetected	1228	RR = 0.96 [0.90, 1.01]	Low
Rate of BCS (T+L vs. T)	4	No	No	No	Serious	Undetected	817	RR = 1.01 [0.88, 1.15]	Moderate
Rate of BCS (L vs. T)	6	No	No	No	Serious	Undetected	1496	RR = 0.94 [0.86, 1.04]	Moderate
CHF (T+L vs. T)	7	Serious	Serious	No	Serious	Undetected	9453	RR = 0.95 [0.73, 1.23]	Very low
CHF (L vs. T)	9	Serious	No	No	Serious	Detected	5893	RR = 0.89 [0.62, 1.28]	Very low
Decline of LVEF (T+L vs. T)	7	Serious	Serious	No	Serious	Undetected	9453	RR = 0.82 [0.67, 1.01]	Very low
Decline of LVEF (L vs. T)	9	Serious	No	No	No	Undetected	5890	RR = 0.67 [0.50, 0.90]	Moderate
Diarrhea (T+L vs. T)	8	Serious	Serious	No	No	Undetected	9512	RR = 8.32 [6.49, 10.68]	Low
Diarrhea (L vs. T)	11	Serious	Serious	No	No	Undetected	6488	RR = 5.62 [4.41, 7.17]	Low
Diarrhea (L vs. T, I-III)	9	Serious	No	No	No	Undetected	5866	RR = 7.90 [5.88, 10.62]	Moderate
Diarrhea (L vs. T, MBC)	2	Serious	No	No	Serious	Undetected	622	RR = 0.99 [0.46, 2.15]	Low
Diarrhea (L vs. T, neoadjuvant therapy)	8	Serious	No	No	No	Undetected	1733	RR = 6.97 [4.46, 10.91]	Moderate
Diarrhea (L vs. T, palliative therapy)	2	Serious	No	No	Serious	Undetected	622	RR = 0.99 [0.46, 2.15]	Low
Neutropenia (T+L vs. T)	5	Serious	No	No	Serious	Undetected	906	RR = 1.16 [0.86, 1.56]	Low
Neutropenia (L vs. T)	8	Serious	Serious	No	Serious	Undetected	2098	RR = 0.99 [0.89, 1.09]	Very low
Fatigue (T+L vs. T)	4	Serious	No	No	Serious	Undetected	628	RR = 0.84 [0.42, 1.67]	Low
Fatigue (L vs. T)	6	Serious	No	No	Serious	Undetected	1722	RR = 1.44 [0.97, 2.11]	Low
Rash/Skin toxicity (T+L vs. T)	6	Serious	No	No	No	Undetected	9357	RR = 6.75 [4.66, 9.77]	Moderate
Rash/Skin toxicity (L vs. T)	5	Serious	No	No	No	Undetected	6352	RR = 8.71 [5.64, 13.45]	Moderate
Vomiting (T+L vs. T)	4	Serious	No	No	Serious	Undetected	599	RR = 2.17 [0.91, 5.19]	Low
Vomiting (L vs. T)	6	Serious	No	No	Serious	Undetected	1718	RR = 1.29 [0.69, 2.43]	Low
Nausea (T+L vs. T)	4	Serious	No	No	Serious	Undetected	599	RR = 1.61 [0.64, 4.06]	Low
Nausea (L vs. T)	6	Serious	No	No	Serious	Detected	1718	RR = 1.02 [0.58, 1.80]	Very low

## Discussion

This is an updated systematic review and meta-analysis, which conclusively determined whether efficacies of trastuzumab plus lapatinib or lapatinib therapy are not inferior to trastuzumab therapy. In previous studies, Yu et al. and Clavarezza et al. [44, 45] did not compare lapatinib therapy with standard trastuzumab therapy, and the latest included studies were published in 2017. Xu et al. [46] reported the meta-analysis results among three arms (T+L, T, and L). However, this study [46] with the included RCTs from 2012 to 2015 did not include all relevant studies. Moreover, all treatment types (neoadjuvant, adjuvant, or palliative treatment) were included in this study, while it is different from the studies of Ma et al. [47] and Guarneri et al. [48], which only included the RCTs of neoadjuvant therapy. Thus, our study included all relevant RCTs, enlarged the sample size, and almost analyzed all important outcomes, which may lead to a more scientific and comprehensive meta-analysis results. Compared to previous studies, our findings basically showed no significant difference, and most results were highly similar.

This meta-analysis shows that the efficacy of trastuzumab combined with lapatinib therapy is superior to standard trastuzumab therapy alone, with a significant improvement in OS, DFS/EFS, pCR (ypT0/is ypN0), and pCR (ypT0/is ypN0/+), RFS, but has more safety risks, with a higher incidence of diarrhea and rash/skin toxicity. In addition, standard trastuzumab therapy alone was proven superior to lapatinib therapy in efficacy, with a significant improvement in OS, PFS, DFS/EFS, pCR (ypT0/is ypN0), and pCR (ypT0/is ypN0/+). With regard to safety, standard trastuzumab therapy alone had a higher incidence of LVEF decline, but had a low incidence of grade III or IV diarrhea and rash/skin toxicity, compared to lapatinib therapy alone. In previous studies, Clavarezza et al. [45] reported that trastuzumab combined with lapatinib therapy significantly increased the pCR rate, compared to trastuzumab therapy alone, in tandem with our findings. Xu et al. [46] reported that trastuzumab combined with lapatinib therapy significantly improved the pCR, EFS, and OS, but showed a higher rate of grade III/IV diarrhea, rash or erythema, and neutropenia, compared to lapatinib or trastuzumab therapy alone, which is in tandem with our findings. Ma et al. [47] reported that standard trastuzumab therapy alone plus chemotherapy was superior to chemotherapy plus lapatinib therapy in pCR (ypT0/is ypN0/+) (RR=0.82, 95% CI: 0.72–0.93) and pCR (ypT0/is ypN0) (RR=0.77, 95% CI: 0.67–0.88), while lapatinib plus trastuzumab therapy and lapatinib therapy showed no significant difference in rate of BCS compared with chemotherapy plus trastuzumab therapy, and lapatinib plus trastuzumab therapy and lapatinib therapy showed higher incidence of diarrhea and skin rash compared with chemotherapy plus trastuzumab therapy. Guarneri et al. [48] reported that trastuzumab combined with lapatinib therapy significantly improved RFS and OS, compared to standard trastuzumab therapy.

OS, PFS, and DFS/EFS are important clinical outcomes. We found that trastuzumab combined with lapatinib therapy significantly improved OS and DFS/EFS, compared to standard trastuzumab therapy alone while lapatinib plus chemotherapy had a lower efficacy in OS, PFS, and DFS/EFS, compared to standard trastuzumab therapy. Based on GRADE, evidence quality for these outcomes was generally moderate, except DFS/EFS (L vs. T), which was considered low quality. Therefore, trastuzumab plus lapatinib is a better option for increasing the survival time of patients. Although lapatinib plus trastuzumab therapy had higher pCR rates, when compared to standard trastuzumab therapy, while trastuzamab was superior to lapatinib, differences among the three kinds of anti-HER2 therapy with regard to breast-conserving rate were insignificant. Thus, we consider treatment of better pCR efficacy may have no obvious clinical meaning for patients who think highly of breast conservation. However, if patients with early breast cancer think highly of short-term efficacy, then trastuzumab combined with lapatinib may be a better choice. We also established that patients with better pCR efficacies had better long-term survival outcomes. However, it has yet to be established whether pCR is associated with long-term survival outcomes.

Trastuzumab-associated cardiac toxicities have been evaluated. Some studies reported that trastuzumab-induced cardiotoxicity might result from its negative regulation of murine double minute 2 (MDM2) and p53. Meanwhile, trastuzumab-induced cardiomyocyte apoptosis has been associated with inflammatory infiltrations. Chemokine expressions of TNFα, MCP-1 and ICAM-1 mediated by TLR4 contribute to the inflammatory responses. Lapatinib preserved cell energy and inhibited TNFα-induced cardiomyocyte apoptosis by activating the AMPK pathway [49, 50]. In this study, lapatinib showed a lower incidence of LVEF decline, compared to trastuzumab therapy, and evidence quality was moderate. Therefore, for patients with bad cardiac conditions, the efficacies of a combination of lapatinib with trastuzumab should be evaluated. With regard to other toxicities, trastuzumab had a lower incidence of grade III/IV diarrhea and rash/skin toxicity, compared to lapatinib therapy and lapatinib plus trastuzumab therapy. Mayo et al. reported that lapatinib can reduce gut microbial diversity, which may be the reason for the high incidence of diarrhea [51]. However, high incidences of rash during treatment with lapatinib and combination therapy may not be a bad thing. Amir Sonnenblick reported that patients with early development of rash derive superior benefits from lapatinib-based therapies [52]. However, reasons for the rash remain unclear. Researchers inferred that lapatinib pharmacokinetics or pharmacodynamics influenced rash development. Normal epidermal growth depends on EGFR, which is expressed on the proliferating skin [52].

In previous studies [44–48], no evidence quality assessment was performed. To determine the reliability of the meta-analysis results, GRADE was used to assess the evidence quality in this study. In long-term survival outcomes (excluding subgroup analysis results), almost all outcomes were assessed moderate quality evidence due to unclear allocation concealment of the included studies, except DFS/EFS (L vs. T) and RFS (T+L vs. T), which were assessed very low evidence quality. Thus, we supposed it was generally credible that lapatinib plus trastuzumab therapy had a better long-term efficacy, when compared to standard trastuzumab therapy, while trastuzamab was superior to lapatinib. In short-term survival outcomes, more than half of the outcomes were assessed low- or very low-quality evidence due to unclear allocation concealment of the included studies, high heterogeneity, or insufficient sample size. Although this study showed that lapatinib plus trastuzumab therapy had a better short-term efficacy, when compared to standard trastuzumab therapy, while trastuzamab was superior to lapatinib, it is still hard to make a conclusion that which therapy had a better short-term efficacy, while outcomes of rate of BCS were assessed moderate-quality evidence due to insufficient sample size. This study proved that no significant difference was found in rate of BCS among three therapies, which, we suppose, was credible. In cardiac toxicities and other toxicities (excluding subgroup analysis results), almost all outcomes were assessed low- or very low-quality evidence due to unclear allocation concealment of the included studies, high heterogeneity, insufficient sample size, or publication bias. Thus, further verification is needed to determine which therapy is safer. Overall, no outcomes had high-quality evidence, 15 outcomes had moderate-quality evidence, 14 outcomes had low-quality evidence, and 8 outcomes had very low-quality evidence. More than half of the outcomes were assessed low- or very low-quality evidence. We inferred that it was probably caused by the following reasons. First, most included studies did not design well, which caused serious risk bias. Second, insufficient sample size led to insignificant differences in some results, which caused serious imprecision. Third, publication bias and high heterogeneity downgraded the level of evidence. To upgrade the evidence quality, more well-designed long-term large sample RCTS are needed. Apart from that, more strict inclusion and exclusion criteria should be made in future studies, so that more studies with low heterogeneity can be included.

To determine the source of heterogeneity, subgroup analysis revealed subgroup effects between groups. Patients with neoadjuvant therapy were associated with longer OS, relative to patients with MBC, while patients with stage I–III breast cancer or neoadjuvant therapy had higher incidences of diarrhea than patients with MBC or palliative therapy during lapatinib treatment. Outcomes from subgroup analyses may have been affected by the instability caused by small sample sizes. We also assessed the quality of evidence and found moderate-quality evidence for OS (L vs. T, palliative therapy), diarrhea (L vs, T, I-III), and diarrhea (L vs. T, neoadjuvant therapy), and low-quality evidence for OS (L vs. T, neoadjuvant therapy), diarrhea (L vs, T, MBC), and diarrhea (L vs. T, palliative therapy), which may inform clinicians and patients when selecting treatment options.

In recent years, studies have increasingly evaluated the efficacies of dual-targeted therapy versus single-targeted therapy. It is significant for clinicians and patients to evaluate the efficacy and safety of dual and single-targeted therapy for better therapeutic selection. This study is associated with various limitations; first, most of the included studies did not clearly mention allocation concealment, which reduces the reliability of the included studies. Second, different chemotherapies in the included studies may lead to clinical heterogeneity. Third, most of the trials, apart from ALTTO, had small sample sizes. Finally, low incidences of safety events in the studies may have led to excess judgment of treatment effects. In the future, relevant, well-designed long-term large sample RCTS are needed, and more studies should assess the mechanisms of cardiac and non-cardiac toxicities of lapatinib and trastuzumab. In addition, it is of significance to determine whether pCR has any effects on long-term survival or not when lapatinib and trastuzumab are used, and whether combinations of lapatinib and trastuzumab can reduce incidences of cardiac toxicities.

## Conclusions

The efficacy of trastuzumab combined with lapatinib therapy is superior to standard trastuzumab therapy alone, but has more non-cardiac grade III/IV toxicities. The efficacy of lapatinib therapy is inferior to that of standard trastuzumab therapy alone. However, the cardiac safety of lapatinib therapy is superior to that of standard trastuzumab therapy.

## Supplementary Information

**Additional file 1: Figure S1**. Subgroup analysis of OS in accordance with tumor stage (L vs. T). **Figure S2**. Subgroup analysis of OS in accordance with therapy setting (T+L vs. T). **Figure S3**. Subgroup analysis of OS in accordance with therapy setting (L vs. T). **Figure S4**. Subgroup analysis of OS in accordance with hormone status (L vs. T). **Figure S5**. Subgroup analysis of DFS/EFS in accordance with therapy setting (T+L vs. T). **Figure S6**. Subgroup analysis of DFS/EFS in accordance with hormone status (T+L vs. T). **Figure S7**. Subgroup analysis of DFS/EFS in accordance with hormone status (L vs. T). **Figure S8**. Subgroup analysis of pCR(ypT0/is ypN0) in accordance with hormone status (T+L vs. T). **Figure S9**. Subgroup analysis of pCR(ypT0/is ypN0) in accordance with hormone status (L vs. T). **Figure S10**. Subgroup analysis of pCR(ypT0/is ypN0/+) in accordance with hormone status (T+L vs. T). **Figure S11**. Subgroup analysis of pCR(ypT0/is ypN0/+) in accordance with hormone status (L vs. T). **Figure S12**. Subgroup analysis of CHF in accordance with therapy setting (T+L vs. T). **Figure S13**. Subgroup analysis of decline of LVEF in accordance with therapy setting (T+L vs. T). **Figure S14**. Subgroup analysis of diarrhea in accordance with tumor stage (L vs. T). **Figure S15**. Subgroup analysis of diarrhea in accordance with therapy setting (T+L vs. T). **Figure S16**. Subgroup analysis of diarrhea in accordance with therapy setting (L vs. T). **Figure S17**. Subgroup analysis of neutropenia in accordance with tumor stage (L vs. T). **Figure S18**. Subgroup analysis of rash/skin toxicity in accordance with tumor stage (L vs. T). **Figure S19**. Subgroup analysis of rash/skin toxicity in accordance with therapy setting (T+L vs. T). **Figure S20**. Subgroup analysis of rash/skin toxicity in accordance with therapy setting (L vs. T).

**Additional file 2: Figure S1**. Egger’s test of OS (T+L vs. T). **Figure S2**. Egger’s test of OS (L vs. T). **Figure S3**. Egger’s test of DFS/EFS (T+L vs. T). **Figure S4**. Egger’s test of DFS/EFS (L vs. T).

**Additional file 3: Table S1**: Details of included trials

**Additional file 4: Table 2**: Summary of meta-analysis and subgroup analysis results

## Data Availability

The datasets generated during and/or analyzed during the current study are available in the CNKI, Wanfang, VIP, Sinomed, PubMed, Embase, and Cochrane CENTRAL databases.
