# Supplementary material for: Lapatinib and lapatinib plus trastuzumab therapy versus trastuzumab therapy for HER2 positive breast cancer patients: an updated systematic review and meta-analysis
Source: Syst Rev. 2022 Dec 10;11:264. doi: 10.1186/s13643-022-02134-9 (PMC9738024; doi:10.1186/s13643-022-02134-9)
Supplement: Supplementary file 1 — Additional file 1: Figure S1. Subgroup analysis of OS in accordance with tumor stage (L vs. T). Figure S2. Subgroup analysis of OS in accordance with therapy setting (T+L vs. T). Figure S3. Subgroup analysis of OS in accordance with therapy setting (L vs. T). Figure S4. Subgroup analysis of OS in accordance with hormone status (L vs. T). Figure S5. Subgroup analysis of DFS/EFS in accordance with therapy setting (T+L vs. T). Figure S6. Subgroup analysis of DFS/EFS in accordance with hormone status (T+L vs. T). Figure S7. Subgroup analysis of DFS/EFS in accordance with hormone status (L vs. T). Figure S8. Subgroup analysis of pCR(ypT0/is ypN0) in accordance with hormone status (T+L vs. T). Figure S9. Subgroup analysis of pCR(ypT0/is ypN0) in accordance with hormone status (L vs. T). Figure S10. Subgroup analysis of pCR(ypT0/is ypN0/+) in accordance with hormone status (T+L vs. T). Figure S11. Subgroup analysis of pCR(ypT0/is ypN0/+) in accordance with hormone status (L vs. T). Figure S12. Subgroup analysis of CHF in accordance with therapy setting (T+L vs. T). Figure S13. Subgroup analysis of decline of LVEF in accordance with therapy setting (T+L vs. T). Figure S14. Subgroup analysis of diarrhea in accordance with tumor stage (L vs. T). Figure S15. Subgroup analysis of diarrhea in accordance with therapy setting (T+L vs. T). Figure S16. Subgroup analysis of diarrhea in accordance with therapy setting (L vs. T). Figure S17. Subgroup analysis of neutropenia in accordance with tumor stage (L vs. T). Figure S18. Subgroup analysis of rash/skin toxicity in accordance with tumor stage (L vs. T). Figure S19. Subgroup analysis of rash/skin toxicity in accordance with therapy setting (T+L vs. T). Figure S20. Subgroup analysis of rash/skin toxicity in accordance with therapy setting (L vs. T). [file 13643_2022_2134_MOESM1_ESM.docx]

**Subgroup analysis:**

**1. OS**

Subgroups were divided in accordance with tumor stage of I-III or MBC. The L arm shows no statistical significance of OS in I-III patients, compared to T arm (HR: 1.05, 95% CI: 0.76-1.47 *p* =0.76; Figure S1). The L arm shows no statistical significance in OS of MBC patients, compared to T arm (HR: 1.40, 95% CI: 1.10-1.80 *p* =0.007; Figure S1). No subgroup differences were found (interaction test, *p* = 0.18).

Subgroups were divided in accordance with type of treatment including neoadjuvant, adjuvant and palliative therapy. The T+L arm shows no statistical significance in OS of patients with adjuvant therapy, compared to T arm (HR: 0.87, 95% CI: 0.75-1.01 *p* =0.06; Figure S2). The T+L arm shows no statistical significance in OS of patients with neoadjuvant therapy, compared to T arm (HR: 0.64, 95% CI: 0.39-1.06 *p* =0.09; Figure S2). No subgroup differences were found (interaction test, *p* = 0.27). The L arm shows no statistical significance in OS of patients with neoadjuvant therapy, compared to T arm (HR: 0.85, 95% CI: 0.60-1.20 *p* =0.36; Figure S3). The L arm shows no statistical significance in OS of patients with palliative therapy, compared to T arm (HR: 1.40, 95% CI: 1.10-1.80 *p* =0.007; Figure S3). Subgroup differences were found (interaction test, *p* = 0.02).

Subgroups were divided in accordance with hormone status. The L arm shows no statistical significance in OS of HR+ patients, compared to T arm (HR: 0.85, 95% CI: 0.60-1.20 *p* =0.36; Figure S4). The L arm shows no statistical significance in OS of HR- patients, compared to T arm (HR: 1.05, 95% CI: 0.65-1.71 *p* =0.36; Figure S4). No subgroup differences were found (interaction test, *p* = 0.16).

**2. DFS/EFS**

Subgroups were divided in accordance with type of treatment including adjuvant and neoadjuvant therapy. The T+L arm shows statistically significant improvement in DFS/EFS of patients with adjuvant therapy, compared to T arm (HR: 0.90, 95% CI: 0.81-1.00 *p*=0.04; Figure S5). The T+L arm shows no statistical significance in DFS/EFS of patients with neoadjuvant therapy, compared to T arm (HR: 0.67, 95% CI: 0.39-1.17 *p* =0.16; Figure S5). No subgroup differences were found (interaction test, *p* = 0.31).

Subgroups were divided in accordance with hormone receptor status. The T+L arm shows no statistical significance in DFS/EFS of HR positive patients, compared to T arm (HR: 0.90, 95% CI: 0.78-1.03 *p* =0.14; Figure S6). The T+L arm shows no statistical significance in DFS/EFS of HR negative patients, compared to T arm (HR: 0.88, 95% CI: 0.76-1.02 *p* =0.10; Figure S6). No subgroup differences were found (interaction test, *p* = 0.84). Meanwhile, subgroups were divided in accordance with hormone receptor status between L and T arms. The L arm shows no statistical significance in DFS/EFS of HR positive patients, compared to T arm (HR: 0.87, 95% CI: 0.58-1.30 *p* =0.49; Figure S7). The L arm shows no statistical significance in DFS/EFS of HR negative patients, compared to T arm (HR: 1.17, 95% CI: 0.82-1.67 *p* =0.40; Figure S7). No subgroup differences were found (interaction test, *p* = 0.28).

**3. pCR (ypT0/is ypN0)**

Subgroups were divided in accordance with hormone receptor status among T+L, L and T arms. The T+L arm shows no statistical significance in pCR (ypT0/is ypN0) of HR positive patients, compared to T arm (RR: 1.15, 95% CI: 0.95-1.38 *p*=0.15; Figure S8). The T+L arm shows statistically significant improvement in pCR (ypT0/is ypN0) of HR negative patients, compared to T arm (RR: 1.22, 95% CI: 1.03-1.45 *p*=0.02; Figure S8). No subgroup differences were found (interaction test, *p* = 0.63). The L arm shows no statistical significance in pCR (ypT0/is ypN0) of HR positive patients, compared to T arm (RR: 0.82, 95% CI: 0.65-1.04 *p*=0.10; Figure S9). The L arm shows no statistical significance in pCR (ypT0/is ypN0) of HR negative patients compared with T arm, compared to T arm (RR: 0.83, 95% CI: 0.66-1.04 *p*=0.06; Figure S9). No subgroup differences were found (interaction test, *p* = 0.94).

**4. pCR (ypT0/is ypN0/+)**

Subgroups were divided in accordance with hormone receptor status among T+L, L and T arms (31, 35, 41, 43). The T+L arm shows no statistical significance in pCR (ypT0/is ypN0/+) of HR positive patients, compared to T arm (RR: 1.20, 95% CI: 0.95-1.51 *p*=0.12; Figure S10). The T+L arm shows statistically significant improvement in pCR (ypT0/is ypN0/+) of HR negative patients, compared to T arm (RR: 1.34, 95% CI: 1.11-1.63 p=0.002; Figure S10). No subgroup differences were found (interaction test, *p* = 0.45). The L arm shows no statistical significance in pCR (ypT0/is ypN0/+) of HR positive patients, compared to T arm (RR: 0.95, 95% CI: 0.76-1.18 *p*=0.63; Figure S11). The L arm shows no statistical significance in pCR (ypT0/is ypN0/+) of HR negative patients, compared to T arm (RR: 0.7, 95% CI: 0.71-1.07 *p*=0.19; Figure S11). No subgroup differences were found (interaction test, *p* = 0.59).

**5. CHF**

Subgroups were divided in accordance with type of treatment including adjuvant therapy and neoadjuvant therapy. The T+L arm shows no statistical significance in CHF of patients with adjuvant therapy, compared to T arm (RR: 0.96, 95% CI: 0.52-1.75 *p*=0.89; Figure S12). The T+L arm shows no statistical significance in CHF of patients with neoadjuvant therapy, compared to T arm (RR: 0.51, 95% CI: 0.03-9.25 *p*=0.65; Figure S12). No subgroup differences were found (interaction test, *p* = 0.68).

**6. Decline of LVEF**

Subgroups were divided in accordance with type of treatment including adjuvant therapy and neoadjuvant therapy. The T+L arm shows no statistical significance in decline of LVEF of patients with adjuvant therapy, compared to T arm (RR: 0.80, 95% CI: 0.44-1.44 *p*=0.45; Figure S13). The T+L arm shows no statistical significance in decline of LVEF in patients with neoadjuvant therapy, compared to T arm (RR: 0.57, 95% CI: 0.11-2.97 *p*=0.50; Figure S13). No subgroup differences were found (interaction test, *p* = 0.70).

**7. Diarrhea**

Subgroups were divided in accordance with tumor stage of I-III (24, 27, 29, 31, 33, 37, 41, 43-44) or MBC (38, 39). The L arm shows higher incidence of grade III/IV diarrhea in stage I-III patients, compared to T arm (RR: 7.90, 95% CI: 5.88-10.62 *p*<0.00001; Figure S14). The L arm shows no statistical significance of grade III/IV diarrhea in MBC patients, compared to T arm (RR: 0.99, 95% CI: 0.46-2.15 *p*=0.99; Figure S14). Subgroup differences were found (interaction test, *p*<0.00001).

Subgroups were divided in accordance with type of treatment including neoadjuvant therapy (24, 29, 31, 33, 37, 41, 43-44) and palliative therapy (38, 39). The T+L arm shows higher incidence of grade III/IV diarrhea in patients with adjuvant therapy, compared to T arm (RR: 6.62, 95% CI: 2.09-20.94 *p*=0.001; Figure S15). The T+L arm shows higher incidence of grade III/IV diarrhea in patients with neoadjuvant therapy, compared to T arm (RR: 10.56, 95% CI: 6.12-18.23 *p*<0.00001; Figure S15). No subgroup differences were found (interaction test, *p*=0.47). The L arm shows higher incidence of grade III/IV diarrhea in patients with neoadjuvant therapy, compared to T arm (RR: 6.97, 95% CI: 4.46-10.91 *p*<0.00001; Figure S16). The L arm shows no statistical significance of grade III/IV diarrhea in patients with palliative therapy, compared to T arm (RR: 0.99, 95% CI: 0.46-2.15 *p*=0.99; Figure S16). Subgroup differences were found (interaction test, *p*<0.00001).

**8. Neutropenia**

Subgroups were divided in accordance with tumor stage of I-III or MBC. The L arm shows no statistical of grade III/IV neutropenia in stage I-III patients, compared to T arm (RR: 1.20, 95% CI: 0.77-1.88 *p*=0.42; Figure S17). The L arm shows no statistical significance of grade III/IV neutropenia in MBC patients, compared to T arm (RR: 0.58, 95% CI: 0.27-1.26 *p*=0.17; Figure S17). No subgroup differences were found (interaction test, *p*=0.11).

Subgroups were divided in accordance with type of treatment including neoadjuvant therapy and palliative therapy. The data of Neutropenia (L vs. T, neoadjuvant therapy) and Neutropenia (L vs. T, palliative therapy) is totally same with data of Neutropenia (L vs. T, I-III) and Neutropenia (L vs. T, MBC) (Figure S17).

**9. Rash/Skin toxicity**

Subgroups were divided in accordance with tumor stage of I-III or MBC. The L arm shows higher incidence of grade III/IV rash or skin toxicity in stage I-III patients, compared to T arm (RR: 8.17, 95% CI: 4.82-13.85 *p*<0.00001; Figure S18). The L arm shows no statistical significance of grade III/IV rash or skin toxicity in MBC patients, compared to T arm (RR: 4.86, 95% CI: 0.84-28.03 *p*=0.08; Figure S18). No subgroup differences were found (interaction test, *p*=0.58).

Subgroups were divided in accordance with type of treatment including adjuvant therapy neoadjuvant therapy and palliative therapy. The T+L arm shows higher incidence of grade III/IV rash or skin toxicity in patients with adjuvant therapy, compared to T arm (RR: 7.49, 95% CI: 4.90-11.45, *p*<0.00001; Figure S19). The T+L arm shows higher incidence of grade III/IV rash or skin toxicity in patients with neoadjuvant therapy, compared to T arm (RR: 3.75, 95% CI: 1.68-8.39, *p*=0.001; Figure S19). No subgroup differences were found (interaction test, *p*=0.14). The L arm shows higher incidence of grade III/IV rash or skin toxicity in patients with neoadjuvant therapy, compared to T arm (RR: 6.13, 95% CI: 2.85-13.19, *p*<0.00001; Figure S20). The L arm shows no statistical significance grade III/IV rash or skin toxicity in patients with palliative therapy, compared to T arm (RR: 4.86, 95% CI: 0.84-28.03, *p*=0.08; Figure S20). No subgroup differences were found (interaction test, *p*=0.81).


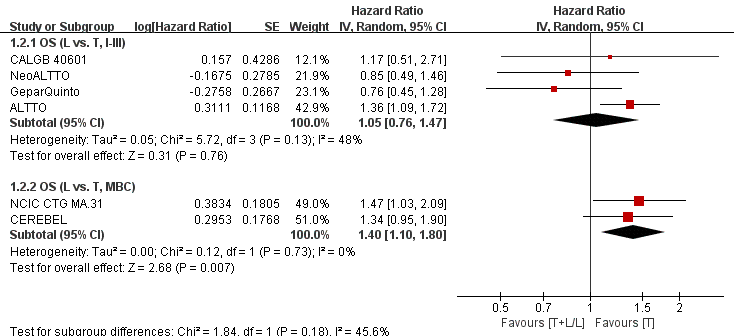


Figure S1. Subgroup analysis of OS in accordance with tumor stage (L vs. T).

**
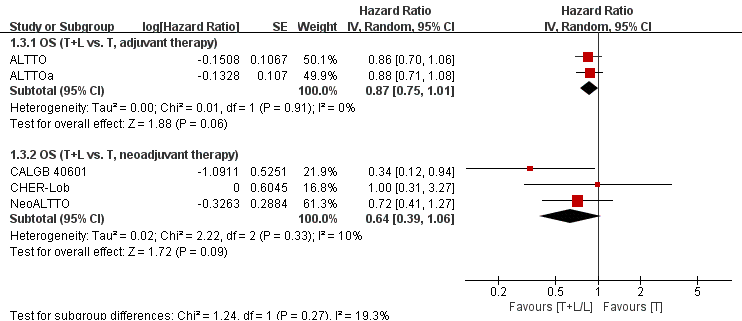
**

Figure S2. Subgroup analysis of OS in accordance with therapy setting (T+L vs. T).


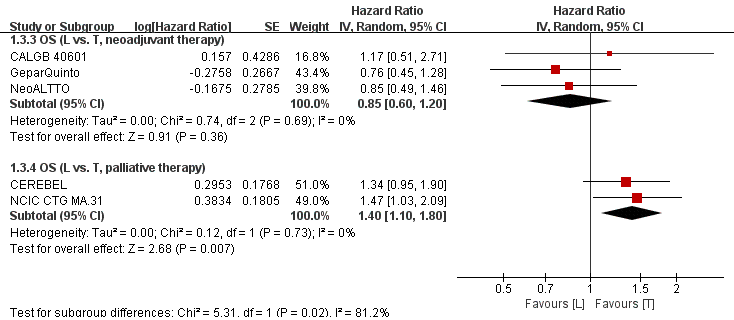


Figure S3. Subgroup analysis of OS in accordance with therapy setting (L vs. T).


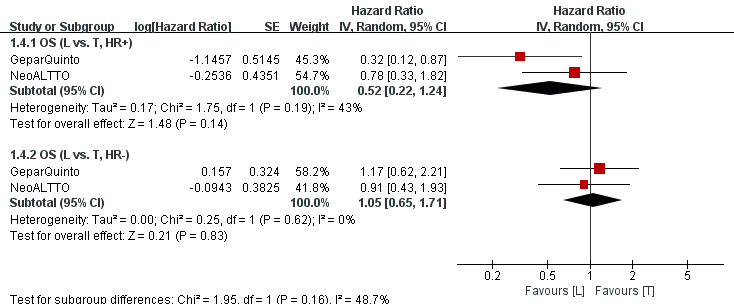


Figure S4. Subgroup analysis of OS in accordance with hormone status (L vs. T).


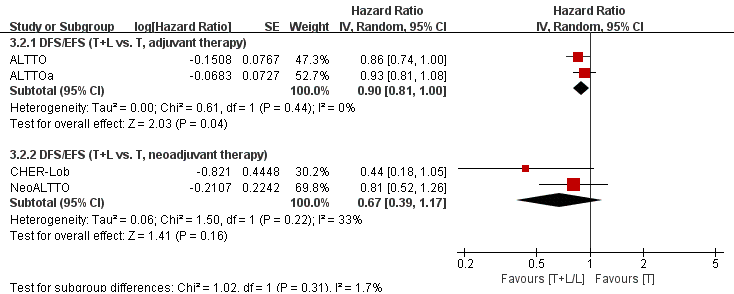


Figure S5. Subgroup analysis of DFS/EFS in accordance with therapy setting (T+L vs. T).

ALTTOa: trastuzumab followed by lapatinib group.


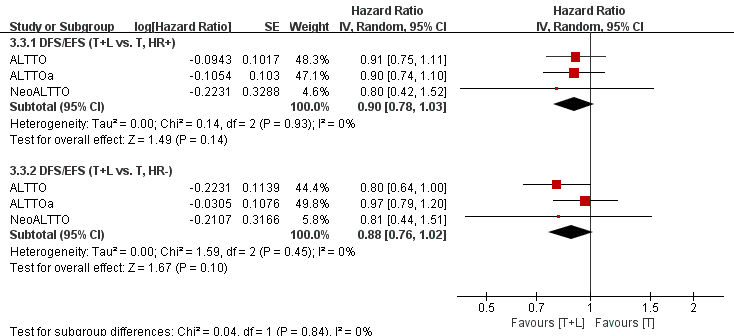


Figure S6. Subgroup analysis of DFS/EFS in accordance with hormone status (T+L vs. T).

ALTTOa: trastuzumab followed by lapatinib group.


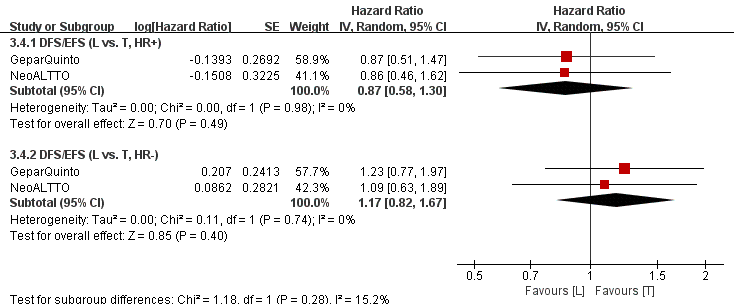


Figure S7. Subgroup analysis of DFS/EFS in accordance with hormone status (L vs. T).


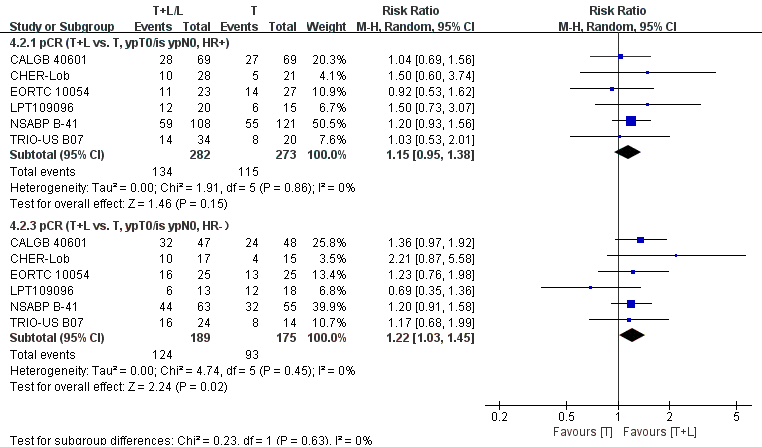


Figure S8. Subgroup analysis of pCR(ypT0/is ypN0) in accordance with hormone status (T+L vs. T).


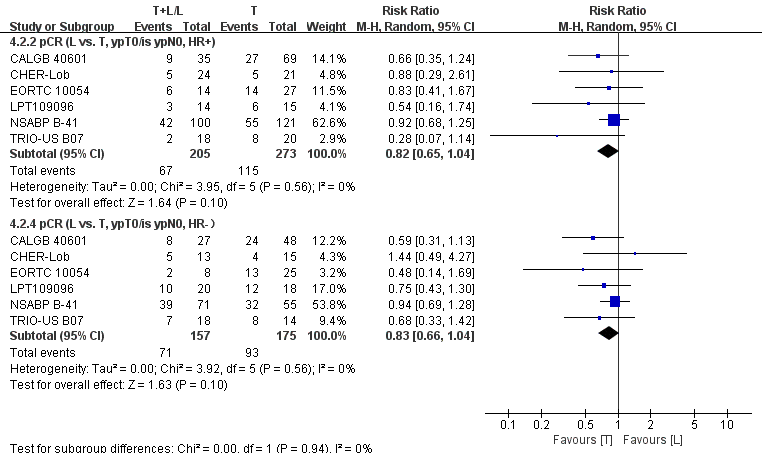


Figure S9. Subgroup analysis of pCR(ypT0/is ypN0) in accordance with hormone status (L vs. T).


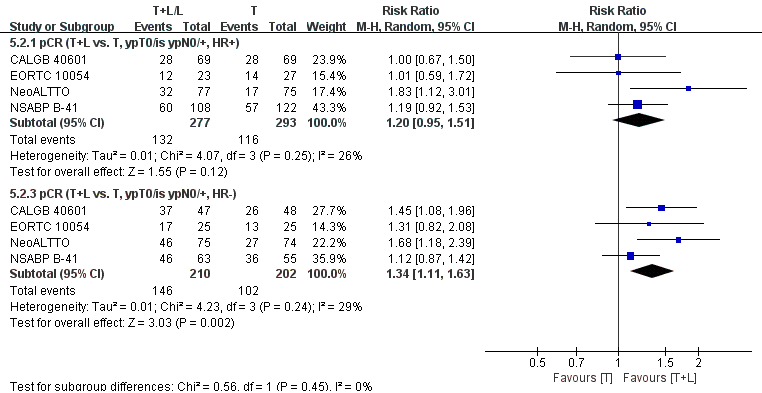


Figure S10. Subgroup analysis of pCR(ypT0/is ypN0/+) in accordance with hormone status (T+L vs. T)


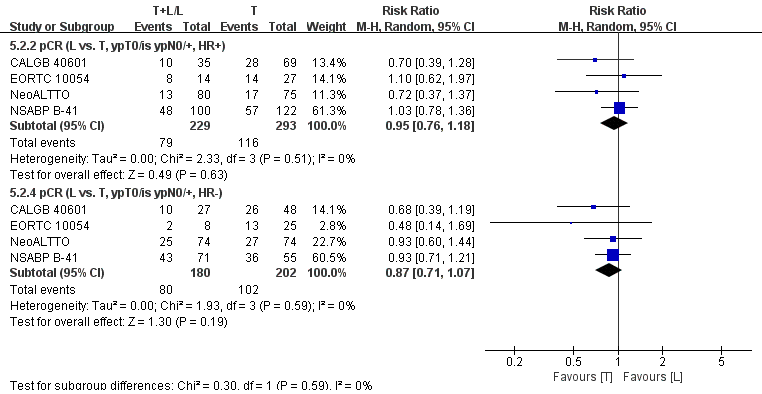


Figure S11. Subgroup analysis of pCR(ypT0/is ypN0/+) in accordance with hormone status (L vs. T).


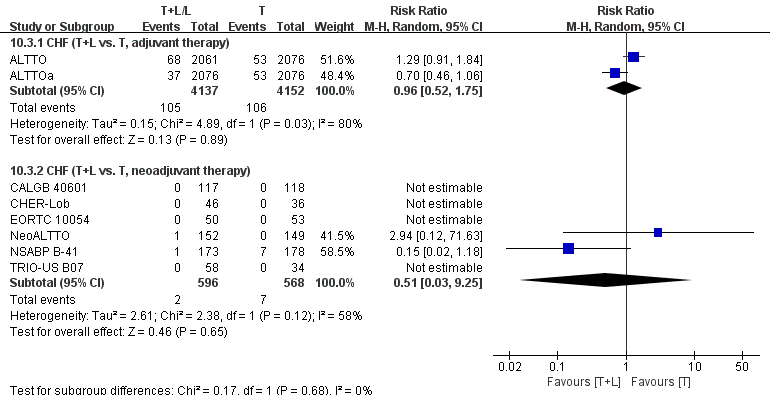


Figure S12. Subgroup analysis of CHF in accordance with therapy setting (T+L vs. T).


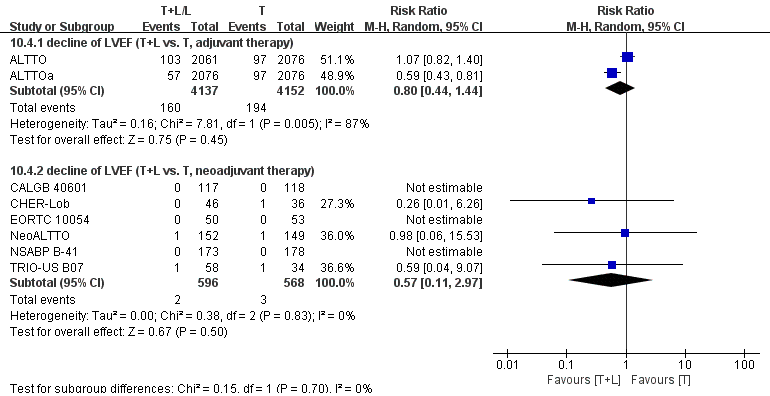


Figure S13. Subgroup analysis of decline of LVEF in accordance with therapy setting (T+L vs. T).


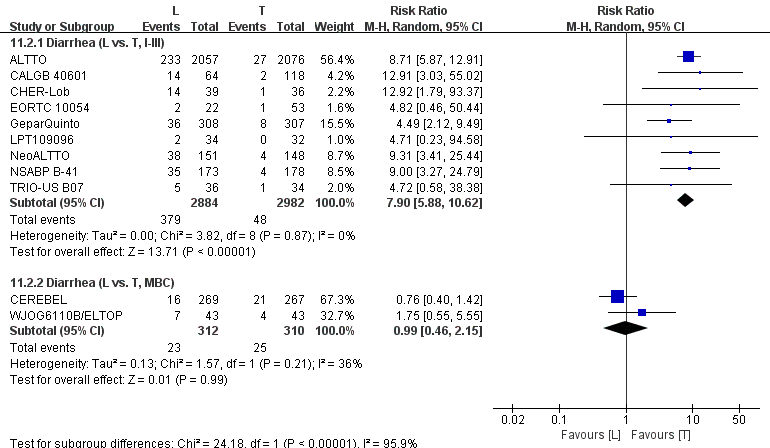


Figure S14. Subgroup analysis of diarrhea in accordance with tumor stage (L vs. T).


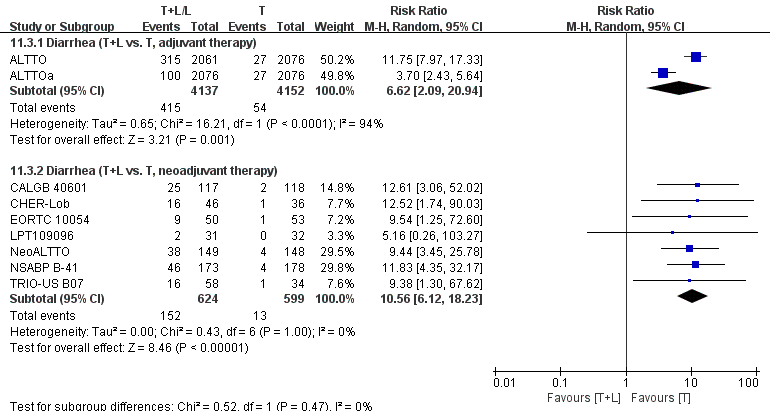


Figure S15. Subgroup analysis of diarrhea in accordance with therapy setting (T+L vs. T).


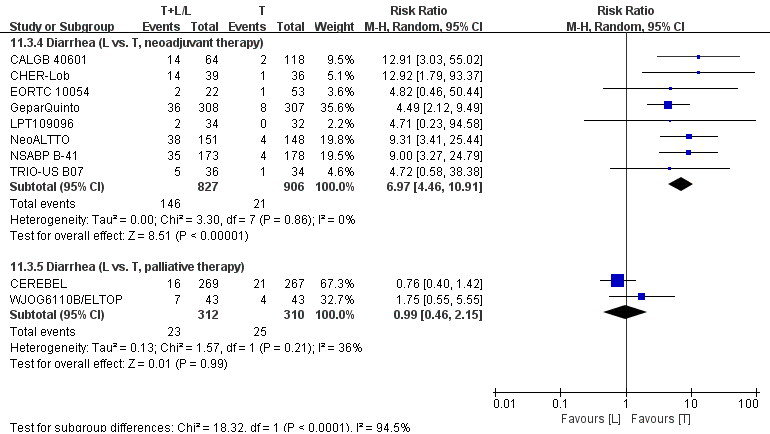


Figure S16. Subgroup analysis of diarrhea in accordance with therapy setting (L vs. T).


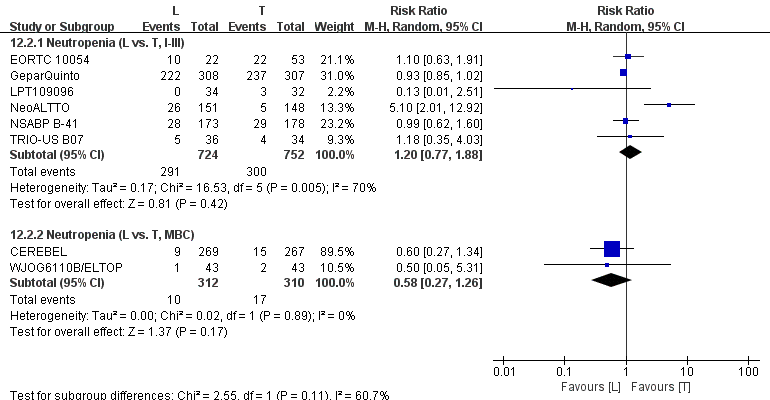


Figure S17. Subgroup analysis of neutropenia in accordance with tumor stage (L vs. T).


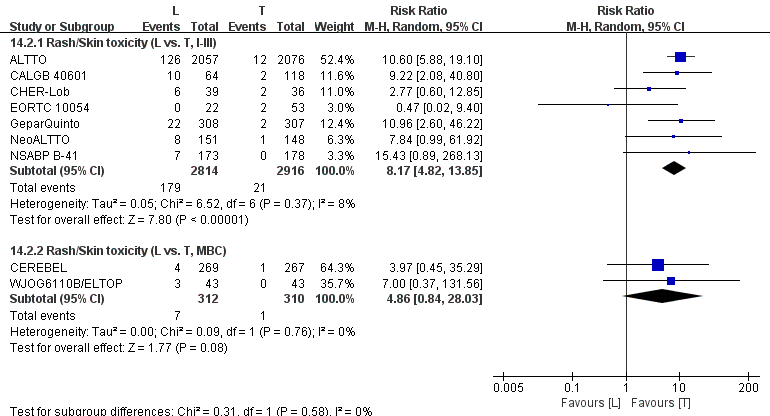


Figure S18. Subgroup analysis of rash/skin toxicity in accordance with tumor stage (L vs. T).


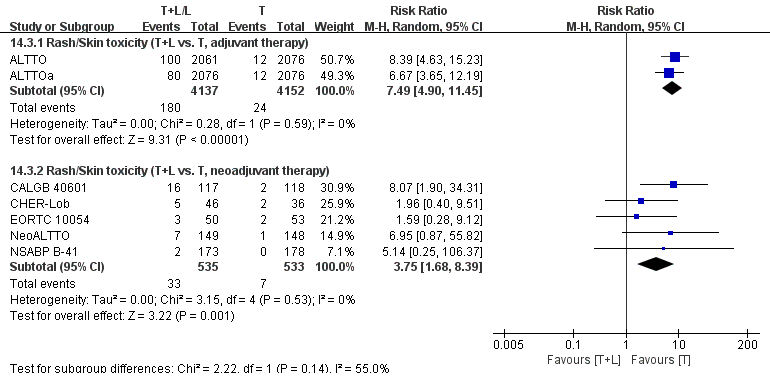


Figure S19. Subgroup analysis of rash/skin toxicity in accordance with therapy setting (T+L vs. T).


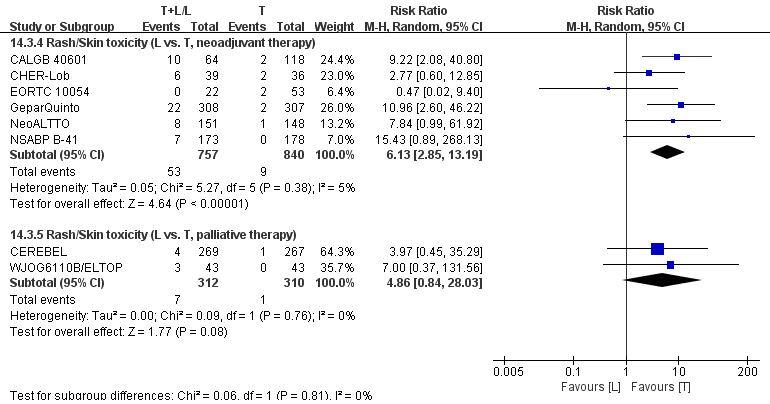


Figure S20. Subgroup analysis of rash/skin toxicity in accordance with therapy setting (L vs. T).
