# Supplementary material for: Lapatinib and lapatinib plus trastuzumab therapy versus trastuzumab therapy for HER2 positive breast cancer patients: an updated systematic review and meta-analysis
Source: Syst Rev. 2022 Dec 10;11:264. doi: 10.1186/s13643-022-02134-9 (PMC9738024; doi:10.1186/s13643-022-02134-9)
Supplement: Supplementary file 2 — Additional file 2: Figure S1. Egger’s test of OS (T+L vs. T). Figure S2. Egger’s test of OS (L vs. T). Figure S3. Egger’s test of DFS/EFS (T+L vs. T). Figure S4. Egger’s test of DFS/EFS (L vs. T). [file 13643_2022_2134_MOESM2_ESM.docx]

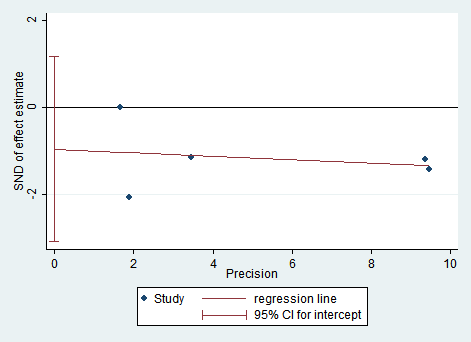


Figure S1. Egger’s test of OS (T+L vs. T).


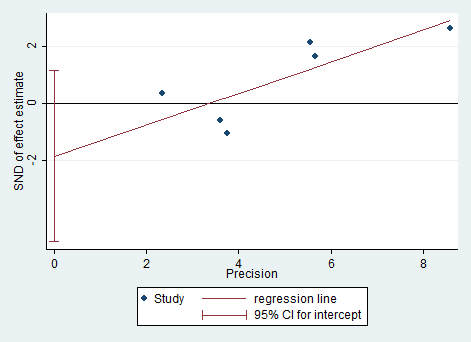


Figure S2. Egger’s test of OS (L vs. T).


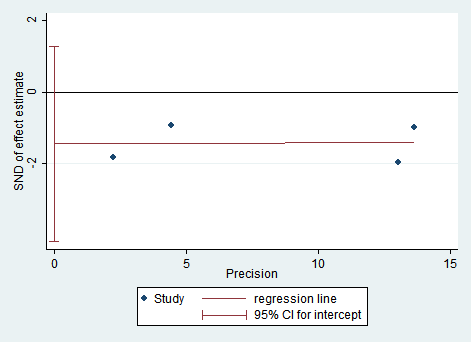


Figure S3. Egger’s test of DFS/EFS (T+L vs. T).


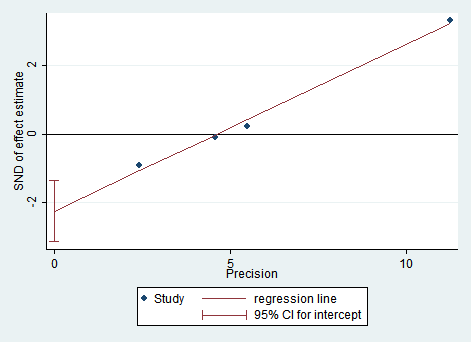


Figure S4. Egger’s test of DFS/EFS (L vs. T).
