# Supplementary material for: Lapatinib and lapatinib plus trastuzumab therapy versus trastuzumab therapy for HER2 positive breast cancer patients: an updated systematic review and meta-analysis
Source: Syst Rev. 2022 Dec 10;11:264. doi: 10.1186/s13643-022-02134-9 (PMC9738024; doi:10.1186/s13643-022-02134-9)
Supplement: Supplementary file 3 — Additional file 3: Table S1: Details of included trials [file 13643_2022_2134_MOESM3_ESM.docx]

| ALTTO | |
| --- | --- |
| Author | Alvaro Moreno-Aspitia 2021  Daniel Eiger 2020  Martine Piccart-Gebhart 2016 |
| Participant | 8381 patients enrolled (L+T: 2093, T→L: 2091, L: 2100, T: 2097)  Median (range) age: T+L arm: 51 (22-80), T→L arm: 51 (22-80), T arm: 51 (18-80)  Median follow up: 6.9 years  Baseline comparability: balanced  Inclusion criteria: Eligible patients were required to have an Eastern Cooperative Oncology Group (ECOG) performance status 0-1, be at least 18 years of age, with clinical or pathologically confirmed stage I to stage III, centrally confirmed HER2 positive (3þ by IHC and/or FISH positive) invasive breast cancer. The hematologic, renal and hepatic functions were to be adequate, and a baseline left ventricular ejection fraction (LVEF) of ≥ 50%, measured by echocardiography or MUGA scan, was required. |
| Intervention | **T+L arm:** participants received treatment per one of three designs. D1: oral lapatinib (OL) 1000 milligrams (mg) daily with trastuzumab (8 milligrams per kilogram [mg/kg] intravenous [IV] loading dose [LD], followed by 6 mg/kg IV every 3 weeks [E3W]) for 52 weeks (wks). D2: OL 750 mg daily plus weekly trastuzumab (4 mg/kg LD, followed by 2 mg/kg IV) concomitantly (conc.) with wkly paclitaxel (pac) 80 mg per squared meter (mg/m^2) IV or docetaxel (doc) 75 mg/m^2 IV E3W for 12 wks. After completion of pac or doc, par. received OL at an increased dose of 1000 mg daily in combination with trastuzumab (6 mg/kg without a LD) E3W for 40 wks. D2B: OL 750 mg plus wkly trastuzumab (4 mg/kg IV LD, followed by 2 mg/kg IV wkly) conc. with doc 75 mg/m^2 E3W and carboplatin (carb) AUC6 IV for 18 wks. After completion of doc and carb, par. received trastuzumab E3W (6 mg/kg without a LD) plus OL 1000 mg daily for 34 wks.  **T→L arm:** participants received treatment per one of the following three designs. Design 1: weekly tras for 12 weeks (4 mg/kg IV loading dose, followed by 2 mg/kg IV weekly), followed by a 6-week washout period, followed by oral lap 1500 mg daily for 34 weeks. Design 2: weekly tras (4 mg/kg IV loading dose, followed by 2 mg/kg IV) concomitantly with weekly paclitaxel 80 mg/m^2 IV or docetaxel 75 mg/m^2 IV every 3 weeks for 12 weeks, followed by a 6-week washout period, followed by oral lap 1500 mg daily for 34 weeks. Design 2B: weekly trastuzumab (4 mg/kg IV loading dose, followed by 2 mg/kg IV weekly) concomitantly with docetaxel 75 mg/m^2 every 3 weeks and carboplatin AUC6 IV for 18 weeks, followed by a 6-week washout period, followed by oral lap 1500 mg daily for 28 weeks  **T arm:** participants received treatment per one of the following three designs. Design 1: trastuzumab 8 mg/kg IV LD, followed by 6 mg/kg IV every 3 weeks for 52 weeks. Design 2: weekly trastuzumab (4 mg/kg IV LD, followed by 2 mg/kg IV weekly) concomitantly with weekly paclitaxel 80 mg/m^2 IV or docetaxel 75 mg/m^2 IV every 3 weeks, for 12 weeks. After completion of paclitaxel or docetaxel, participants received trastuzumab (6 mg/kg without a LD every 3 weeks for 40 weeks. Design 2B: weekly trastuzumab (4 mg/kg IV LD, followed by 2 mg/kg IV weekly) concomitantly with docetaxel 75 mg/m^2 every 3 weeks and carboplatin AUC6 IV, for 18 weeks. After completion of docetaxel and carboplatin, participants received trastuzumab every 3 weeks (6 mg/kg without a LD) for 34 weeks.  **L arm:** participants received treatment per one of the following three designs. Design 1: oral lap 1500 mg daily for 52 weeks. Design 2: oral lap 750 mg daily concomitantly with weekly paclitaxel 80 mg/m^2 IV or docetaxel 75 mg/m^2 IV every 3 weeks, for 12 weeks. After completion of paclitaxel or docetaxel, participants received oral lap at an increased dose of 1500 mg daily for 40 weeks. Design 2B: oral lap 750 mg daily concomitantly with docetaxel 75 mg/m^2 every 3 weeks and carboplatin AUC6 IV, for 18 weeks. After completion of docetaxel and carboplatin, oral lap was given at an increased dose of 1500 mg for 34 weeks. |
| Outcome | 1. DFS (defined as the time from randomization to the recurrence of invasive breast cancer at local, regional or distant sites; contralateral invasive breast cancer; second non-breast malignancy; or death as a result of any cause, whichever occurred first);  2. OS;  3. Safety in general and cardiac safety (Chemotherapy adverse effects were graded according to the National Cancer Institute Common Terminology Criteria for Adverse Events, version 4.0);  4. Time to recurrence;  5. Time to distant recurrence;  6. Time to first brain metastasis. |
| Study design | A prospective randomised, phase 3, open-label, multicenter study;  Clinical trials identifier: NCT00490139. |
| CHER-Lob | |
| Author | Valentina Guarneri 2021  Valentina Guarneri 2015  Valentina Guarneri 2012 |
| Participant | 121 patients enrolled (T+L: 46, L: 39, T: 36)  Mean (range) age: L+T: 48 (26-65), L: 49 (34-68), T: 50 (34-65)  Median follow up: 9 years  Baseline comparability: balanced  Inclusion criteria: previously untreated, infiltrating primary breast cancer of more than 2.0 cm in largest clinical diameter, HER2 positivity (either immunohistochemistry 3+ or fluorescent in situ hybridization amplification), age 18 to 65 years, Eastern Cooperative Oncology Group performance status of 0 to 1, availability of tumor tissue for biologic and molecular examination before starting primary treatment, left ventricular ejection fraction (LVEF) within the institutional range of normal, normal organ and marrow function (leukocytes ≥ 3,000/μL, absolute neutrophil count ≥ 1,500/μL, platelets ≥ 100,000/μL, total bilirubin ≤ 1.5 times the upper limit of normal, and AST and ALT ≤ 2.5 times the upper limit of normal), ability to swallow and retain oral medication, adequate contraception methods for women of childbearing potential, and written informed consent. |
| Intervention | **T+L arm:** trastuzumab 4 mg/kg IV on the day of the first paclitaxel course followed by 2 mg weekly and lapatinib 1000 mg/day orally for 26 weeks prior to surgery  **T arm:** trastuzumab 4mg loading dose followed by 2 mg weekly for 26 weeks prior to surgery  **L arm:** lapatinib 1500 mg/day orally for 26 weeks prior to surgery  All participants received chemotherapy, which included paclitaxel 80 mg/m^2 weekly for 12 weeks, followed by IV fluorouracil 600 mg/m^2, IV epidoxorubicin 75 mg/m^2, and IV cyclophosphamide 600 mg/m^2, once every 21 days for four treatment courses. Trastuzumab or lapatinib was administered throughout the course of the CT and for two weeks after the last CT administration. |
| Outcome | 1. pCR (the absence of residual invasive tumour in breast and axillary nodes.);  2. RFS (calculated from randomisation to BC recurrence (locoregional or distant; contralateral BC excluded) or death from any cause, whichever first.);  3. OS (calculated from randomisation to death from any cause or last follow-up.);  4. The percentage of clinical objective responses in the breast;  5. The percentage of breast-conserving surgery;  6. Conversion rate from mastectomy to BCS;  7. The safety profile of these combinations;  8. The time to treatment failure from start of primary therapy;  9. The percentage of inhibition of intermediate and final biomarkers of the proliferative;  10. Apoptosis pathways induced by the different combinations;  11. The correlation between gene expression at diagnosis and pathologicresponse. |
| Study design | A noncomparative, phase IIb, randomized study;  Clinical trials identifier: NCT00429299. |
| TRIO-US B07 | |
| Author | Sara A. Hurvitz 2020 |
| Participant | 128 patients enrolled (T+L: 58, L: 36, T: 44)  Median age: T+L: 47, L: 51, T: 48  Follow up: unkown  Baseline comparability: balanced  Inclusion criteria: prior exposure to chemotherapy, radiation, or endocrine therapy for currently diagnosed invasive or non-invasive breast cancer, any prior radiation therapy to ipsilateral breast or chest wall, history of any other malignancy within the past 5 years (except non-melanoma skin cancer or carcinoma-in-situ of the cervix), pre-existing motor or sensory neuropathy of grade ≥ 2, pre-existing cardiac disease, gastrointestinal condition causing chronic diarrhea requiring active therapy, concurrent infection requiring parenteral antibiotics, metastatic breast cancer, current treatment with ovarian hormonal replacement therapy, or current treatment with any selective estrogen receptor modulators. Pregnant or lactating women were excluded and contraception was required for females of childbearing potential. |
| Intervention | **T+L arm:** lapatinib at a dose of 1000 mg po qd * 21 days + trastuzumab 8 mg/kg iv once → trastuzumab (6 mg/kg IV) * 6 cycles + lapatinib (1000 mg/day orally days 1–21) * 6 cycles + docetaxel (75 mg/ m^2^ IV q3w) and carboplatin (area under the plasma concentration-time curve [AUC] 6 mg/mL/min q3w) * 6 cycles.  **L arm:** lapatinib at a dose of 1000 mg po qd * lapatinib (1000 mg/day orally days 1–21) * 6 cycles + docetaxel (75 mg/ m^2^ IV q3w) and carboplatin (area under the plasma concentration-time curve [AUC] 6 mg/mL/min q3w) * 6 cycles.  **T arm:** trastuzumab 8 mg/kg iv once → trastuzumab (6 mg/kg IV) * 6 cycles + docetaxel (75 mg/ m^2^ IV q3w) and carboplatin (area under the plasma concentration-time curve [AUC] 6 mg/mL/min q3w) * 6 cycles. |
| Outcome | 1. pCR (defined as an absence of viable invasive tumor cells in the breast and examined axillary lymph nodes at the time of definitive surgery);  2. Safety and tolerability (NCI Common Toxicity Criteria for Adverse Events (CTCAE), version 3.0.). |
| Study design | A randomized, multicenter, open-label, three-arm phase II study;  Clinical trials identifier: NCT00769470 |
| CALGB 40601 | |
| Author | Aranzazu Fernandez-Martinez 2020  Lisa A. Carey 2016 |
| Participant | 305 patients enrolled (T+L: 118, L: 67, T: 120)  Median (range) age: T+L: 48 (24-70), L: 48 (25-74), T: 50 (30-75)  Median follow up: 83 months  Baseline comparability: balanced  Inclusion criteria: patients had newly diagnosed, histologically confirmed, untreated clinical stage II to III HER2-positive disease. HER2 positivity was determined locally by immunohistochemistry or fluorescence in situ hybridization according to American Society of Clinical Oncology/College of American Pathology guidelines. Patients were age ≥ 18 years, had tumors ≥ 1 cm in size, and had a pretreatment left ventricular ejection fraction ≥ 50%. Patients with multicentric or bilateral disease were eligible if the target lesion met other eligibility criteria. Surgery was required within 42 days of last dose. Postsurgery, patients were recommended to receive adjuvant chemotherapy with doxorubicin 60 mg/m^2^ plus cyclophosphamide 600 mg/m2administered every 14 to 21 days for four cycles and 36 additional weeks of trastuzumab. |
| Intervention | **T+L arm:** trastuzumab 2 mg/kg IV and paclitaxel 80 mg/m^2 IV over 1 hour once weekly and lapatinib ditosylate 750 mg PO once daily for 16 weeks  **T arm:** trastuzumab 2 mg/kg IV and paclitaxel 80 mg/m^2 IV over 1 hour once weekly for 16 weeks  **L arm:** lapatinib ditosylate 15000 mg PO once daily and paclitaxel 80 mg/m^2 IV over 1 hour once weekly for 16 weeks  Postsurgery, patients were recommended to receive adjuvant chemotherapy with doxorubicin 60 mg/m^2^ plus cyclophosphamide 600 mg/m^2^ administered every 14 to 21 days for four cycles and 36 additional weeks of trastuzumab. |
| Outcome | 1. RFS (defined as the interval from surgery to ipsilateral invasive breast tumor recurrence, regional recurrence, distant recurrence, or death of any cause, whichever occurred first. Patients without an event were censored at the date of the last clinical assessment.);  2. OS (defined as the interval from random assignment to death or last follow-up.);  3. pCR in the breast (defined as the absence of residual invasive carcinoma.);  4. pCR in breast and ipsilateral axillary lymph nodes (defined as no invasive tumor by hematoxylin and eosinstaining in any lymph node);  5. Adverse events. |
| Study design | A randomized phase III trial;  Clinical trials identifier: NCT00770809. |
| NeoALTTO | |
| Author | Jens Huober 2019  Evandro de Azambuja 2014  C. Criscitiello 2013  José Baselga 2012 |
| Participant | 455 patients enrolled (T+L: 152, L: 154, T: 149)  Median (range) age: T+L: 50 (43-59), L: 50 (42-56), T: 49 (44-57)  Median follow up: 6.7 years  Baseline comparability: balanced  Inclusion criteria: Eligible patients had histologically confirmed invasive breast cancer with HER2 overexpression or amplification as per guidelines. HER2 status was assessed locally at participating institutions that had been accredited by our certified laboratory (Vall d’Hebron Institute of Oncology). Participating patients had primary breast tumors greater than 2 cm in diameter measured by either mammography or echography. Patients had to have adequate baseline hepatic, renal, cardiac, and bone marrow function for inclusion. Adequate cardiac function was defined as a baseline left ventricular ejection fraction of 50% or more measured by echocardiography or multiple gate acquisition scan. Patients were not eligible if they had bilateral breast cancer, inflammatory breast cancer, or distant metastases. |
| Intervention | **T+L arm:** Oral lapatinib 1000 mg daily plus trastuzumab 4 mg/kg IV load followed by 2 mg/kg IV weekly for 6 weeks, followed by lapatinib 750 mg daily plus trastuzumab 2 mg/kg IV weekly plus weekly paclitaxel 80 mg/m^2 IV for an additional 12 weeks  **T arm:** Trastuzumab 4 mg/kilograms [kg] IV load followed by 2 mg/kg IV weekly for 6 weeks, followed by trastuzumab plus weekly paclitaxel (80 mg/m^2 IV) for an additional 12 weeks  **L arm:** Oral lapatinib (1500 milligrams [mg] daily) for 6 weeks, followed by lapatinib plus weekly paclitaxel (80 mg per meters squared [mg/m^2]) intravenously (IV) for an additional 12 weeks |
| Outcome | 1. pCR (defined as the absence of invasive tumour cells in the breast at the time of surgery.);  2. pCR (included the absence of invasive cancer in the breast and ipsilateral axillary lymph nodes at surgery (ypT0/is ypN0));  3. EFS (defined as the time from randomisation to the first EFS event. For women who underwent breast cancer surgery (n = 427), EFS events were defined as post-surgery breast cancer relapse, second primary malignancy or death without recurrence. For women who did not undergo breast cancer surgery (n = 28), EFS events were death during clinical follow-up or non-completion of any neoadjuvant investigational product due to disease progression.);  4. OS (defined as the time from randomisation to death from any cause.);  5. Safety and tolerability;  6. Objective tumour response rate ((complete plus partial) at the end of the biological window and at the time of definitive surgery on the basis of physical examination with WHO criteria);  7. Patients with node-negative disease at surgery;  8. Patients having breast-conserving surgery;  9. Rate of conversion to breast-conserving surgery. |
| Study design | A randomised, multicentre, open-label, phase 3 study;  Clinical trials identifier: NCT00553358. |
| GeparQuinto | |
| Author | Michael Untch 2018  Michael Untch 2012 |
| Participant | 615 patients enrolled (L: 308, T: 307)  Median (range) age: L: 50 (21-73), T: 50 (25-74)  Median follow up: 55 months  Baseline comparability: balanced  Inclusion criteria: female patients with unilateral or bilateral primary invasive breast carcinoma were enrolled in the study. Diagnosis of breast cancer had to be confirmed histologically by core biopsy. HER2 status on tumor biopsy had to be positive by either immunohistochemistry (IHC 3+) or in situ hybridization (ratio ≥ 2.0) by the local pathologist. Tumor lesions were required to have a palpable size of ≥ 2 cm or a size of ≥ 1 cm in maximum diameter and measurable in two-dimensions, preferably by sonography. In the case of inflammatory disease, the clinical extent of inflammation was used as measurable lesion. Patients with locally advanced tumors stage cT4 or cT3, hormone receptor–negative tumors (estrogen receptor [ER] and progesterone receptor [PgR], 10%), or hormone receptor–positive tumors (ER and/or PgR $ 10%) with clinically positive axillary nodes (cN+ for cT2 or pNSLN+for cT1) were eligible. |
| Intervention | **L arm:** EC (epirubicin 90 mg/m^2^ and cyclophosphamide 600 mg/m2, day 1, q3w) * 4 cycles + lapatinib 1,250 mg PO qd * 4cycles→ docetaxel (100 mg/m2, day 1, q3w) * 4 cycles + lapatinib 1,250 mg PO qd * 4cycles → surgery → trastuzumab 6mg/kg IV q3w * 52 weeks.  **T arm:** EC (epirubicin 90 mg/m^2^ and cyclophosphamide 600 mg/m2, day 1, q3w) * 4 cycles + trastuzumab 6mg/kg (a loading dose of 8 mg/kg on day 1 of the first EC cycle) IV q3w * 4cycles→ docetaxel (100 mg/m2, day 1, q3w) * 4 cycles + trastuzumab 6mg/kg IV q3w * 4cycles → surgery → trastuzumab 6mg/kg IV q3w * 28 weeks. |
| Outcome | 1. pCR (ypT0 ypN0);  2. DFS (any invasive loco-regional (ipsilateral breast or local/regional lymph nodes) recurrence of disease, any invasive contralateral breast cancer, any distant recurrence of disease, any secondary malignancy, or death as a result of any cause, whichever occurred first.);  3. Distant DFS (DDFS) (any distant recurrence of disease, any secondary malignancy, or death as a result of any cause, whichever occurred first.);  4. Time to loco-regional relapse (TTLRR) (any local or regional (ipsilateral breast [invasive or ductal carcinoma in situ] or local/regional lymph nodes) recurrence of disease, or any invasive contralateral breast cancer, whichever occurred first. Distant metastases, secondary malignancy, or death were considered competing risks.);  5. Time to CNS metastases (TTCNSM) (any CNS metastasis was an event. Other distant metastases, secondary malignancy, or death were considered competing risks.);  6. OS (defined as the time since random assignment until death as a result of any cause.);  7. Toxic effects, compliance (National Cancer Institute Common Terminology Criteria (NCI-CTC) version 3.0.);  8. Response rates of the tumour and axillary nodes by physical examination and imaging tests (sonography, mammography, or MRI) before surgery (defined as no evidence of disease in the breast by ultrasound, or, if ultrasound was not possible, by mammography or physical examination. A partial response was defined as a reduction in the product of the two largest perpendicular diameters of the primary tumour by 50% or more; progressive disease was defi ned as an increase in tumour size by 25% or more or the presence of a new lesion. All remaining scenarios were classified as no change.);  9. Pathological complete response according to different definitions (ypT0 ypN0/+, ypT0/is ypN0; ypT0/is ypN0/+);  10. The breast conservation rate (tumorectomy, segmentectomy, or quadrantectomy). |
| Study design | A randomised phase 3 trial;  Clinical trials identifier: NCT00567554. |
| WJOG6110B/ELTOP | |
| Author | Toshimi Takano 2018 |
| Participant | 86 patients enrolled (L: 43, T: 43)  Median (range) age: L: 59 (37-78), T: 57 (34-81)  Median follow up: 44.6 months  Baseline comparability: balanced  Inclusion criteria: Eligible patients were women aged 20 years or older with HER2-positive MBC or unresectable locally advanced breast cancer who were previously treated with taxanes, with progression on trastuzumab containing regimens. HER2 positivity was defined as 3+ staining by immunohistochemistry or HER2 gene amplification (HER2:CEP1 7 signal ratio of 2.0 or more) by in situ hybridization. Patients treated with more than 2 chemotherapy regimens for MBC were excluded. Eligible patients had an Eastern Cooperative Oncology Group (ECOG) performance status (PS) of 0e2 and adequate bone marrow, cardiac, hepatic, and renal function. Patients with brain metastases were included if they were asymptomatic. |
| Intervention | **T arm:** trastuzumab (4 mg/kg loading then 2 mg/kg weekly or 8 mg/kg loading then 6 mg/kg every 3 weeks) and capecitabine (2500 mg/m2/day on days 1-14 every 3 weeks) until progression or intolerable toxicity.  **L arm:** lapatinib (1250 mg/day) and capecitabine (2000 mg/m2/day on days 1-14 every 3 weeks) until progression or intolerable toxicity. |
| Outcome | 1. Progression-free survival (PFS);  2. Overall survival (OS);  3. The objective response rate (ORR) (Response Evaluation Criteria in Solid Tumors (RECIST) version 1.1.);  4. The disease control rate (DCR) (Response Evaluation Criteria in Solid Tumors (RECIST) version 1.1.);;  5. The proportion of patients with brain metastases as the site of first progression;  6. Safety. |
| Study design | An open-label, multicenter, randomized phase II trial;  Clinical trials identifier: UMIN000005219. |
| CEREBEL | |
| Author | Xavier Pivot 2015 |
| Participant | 540 patients enrolled (L: 271, T: 269)  Median (range) age: L: 53 (27-83), T: 56 (31-79)  Median follow up: unknown  Baseline comparability: balanced  Inclusion criteria: Eligible patients were women ≥ 18 years old with histologically confirmed HER2-positive MBC (HER2 score > 2.2 by fluorescence in situ hybridization and/or 3+ amplification by immunohistochemistry or chromogenic/silver in situ hybridization). No centralized review of HER2 status was undertaken, and testing was performed as per the institution’s local laboratory methods. Patients were required to have received prior anthracycline and/or taxanes for (neo)adjuvant or metastatic disease. Prior trastuzumab was allowed but not required. Eastern Cooperative Oncology Group performance status ≤ 2 and adequate organ function were required. All patients signed an informed consent form. No history of CNS metastases or presence of CNS metastases at baseline was permitted; baseline brain MRI scans were an eligibility screening requirement to exclude potential participants with asymptomatic metastases. |
| Intervention | **L arm:** lapatinib 1,250 mg once daily and capecitabine 2,000 mg/m^2^ per day on days 1 through 14, every 21 days.  **T arm:** a trastuzumab infusion of 6 mg/kg every 3 weeks (with possibly a loading dose of 8 mg/kg on day 1) and capecitabine 2,500 mg/m2per day on days 1 through 14, every 21 days. |
| Outcomes | 1. Incidence of CNS as site of first relapse (based on independent review committee assessment of brain MRI scans. All non-CNS lesions must have qualified as progression of diseaseper RECIST.);  2. Time to first CNS progression (defined as the time from random assignment to the date of first CNS progression);  3. Incidence of CNS progression at any time;  4. PFS (defined as the time from random assignment to the date of disease progression or death from any cause);  5. Overall survival (OS; defined as the time from random assignment to the date of disease progression or death from any cause);  6. Overall response rate (ORR);  7. Duration of response (DoR; time from response until disease progression or death as a result of breast cancer);  8. Safety.  For PFS, time to first CNS progression, and DoR, patients without an event were censored at the last clinical assessment. For OS, patients still alive were censored at the date of last contact. |
| Study design | A phase III, randomized, multicenter, open-label study;  Clinical trials identifier: NCT00820222. |
| NCIC CTG MA.31 | |
| Author | Karen A. Gelmon 2015 |
| Participant | 652 patients enrolled (L: 326, T: 326)  Median age: L: 55.4, T: 54.4  Median follow up: 21.5 months  Baseline comparability: balanced  Inclusion criteria: HER2-positive metastatic BC, Eastern Cooperative Oncology Group performance status 0 to 2, no prior therapy with cytotoxics or biologics for recurrent or advanced disease, baseline left ventricular ejection fraction (LVEF) ≥ 50% (determined by echocardiography or multiple-gated acquisition scanning), measurable or nonmeasurable disease defined by RECIST (version 1.0) criteria, and no major end-organ disease. Prior (neo)adjuvant treatment with anti-HER2 agent and/or taxane was allowed provided the last dose was ≥ 12 months before random assignment. Prior endocrine therapy or radiotherapy was permitted provided ≥ 2 weeks had elapsed from cessation. Brain computed tomography or magnetic resonance imaging was required within 4 weeks before random assignment; patients with brain metastases were ineligible. |
| Intervention | **L arm:** lapatinib po 1250mg qd * 24 weeks + taxane (paclitaxel iv 80mg/m^2^ qw or docetaxel po 75mg/m^2^ q3w) * 24 weeks → lapatinib po 1500mg qd.  **T arm:** 1. Trastuzumab iv 4 mg/kg bolus followed by 2 mg/kg maintenance qw * 24 weeks + paclitaxel iv 80mg/m^2^ qw * 24 weeks → trastuzumab iv 6 mg/kg q3w; 2. Trastuzumab iv 8 mg/kg bolus followed by 6 mg/kg maintenance q3w * 24 weeks + docetaxel po 75mg/m^2^ q3w * 24 weeks → trastuzumab iv 6 mg/kg q3w |
| Outcome | 1. Progression-free survival (PFS) (defined as time from random assignment to disease progression (PD; as assessed by RECIST [version 1.0] criteria) or death.);  2. OS (defined as time from random assignement to death, with censoring at the date of the longest follow-up.);  3. The incidence of and time to magnetic resonance imaging or computed tomography brain metastases at first PD;  4. Overall response rate (ORR; complete [CR] or partial response [PR]);  5. Time to response (from random assignment to date of first CR or PR);  6. Duration of response (from first CR or PR until PD or death);  7. Clinical benefit response (CBR) (defined as the best overall response of CR, PR, or stable disease at 24 weeks.);  8. Adverse event (AE) (National Cancer Institute Common Terminology Criteria for Adverse Events (version 3.0));  9. Quality of life (QOL) (measured by the European Organisation for Research and Treatment of Cancer (EORTC) Quality of Life Questionnaire–Core 30 (QLQ-C30)). |
| Study design | A randomized open-label international phase III trial;  Clinical trials identifier: NCT00667251. |
| EORTC 10054 | |
| Author | H. Bonnefoi 2014 |
| Participant | 128 patients enrolled (T+L: 52 L: 23, T: 53)  Median (range) age: T+L: 49.4 (27.3-70.8), L: 49.9 (27.3-68.5), T: 47 (25.3-68.9)  Median follow up: unknown  Baseline comparability: balanced  Inclusion criteria: age 18 to 70 years, any large operable cT2 or cT3 or locally advanced or inflammatory breast cancer (clinical T4 a, b, c, d, any N or any T, N2 or N3), M0, histologically confirmed diagnosis of invasive breast cancer (patients with bilateral breast cancer were eligible if only one side was HER2-positive), HER2 positive tumor (immunohistochemistry 3+, or IHC 2+ and FISH/CISH +, or FISH or CISH+ only), availability of three tumour biopsies (one fixed in formalin and two frozen) before inclusion, WHO performance status of 0 to 2, adequate baseline cardiac evaluation (including left ventricular ejection fraction(LVEF)measured by echocardiography or multiple gate acquisition scan within the institutional range of normal), hepatic, renal, and bone marrow function. |
| Intervention | **T+L arm:** 3 cycles of docetaxel (100 mg/m²) + trastuzumab weekly schedule + lapatinib (1000 mg/d) followed by 3 cycles of fluorouracil 500 mg/m^2^, epirubicin 100 mg/m^2^, cyclophosphamide 500 mg/m^2.^  **T arm:** 3 cycles of docetaxel (100 mg/m²) + trastuzumab weekly schedule followed by 3 cycles of fluorouracil 500 mg/m^2^, epirubicin 100 mg/m^2^, cyclophosphamide 500 mg/m^2.^  **L arm:** 3 cycles of docetaxel (100 mg/m²) + lapatinib (1000 mg/d) followed by 3 cycles of fluorouracil 500 mg/m^2^, epirubicin 100 mg/m^2^, cyclophosphamide 500 mg/m^2^. |
| Outcome | 1. pCR in the breast (defined as complete disappearance of invasive cancer in the primary tumor with the exception of very few scattered invasive tumor cells (the presence of ductal carcinoma in situ was allowed).);  2. Safety and tolerability of the combination (according to CTCAE 3.0, response rate according to RECIST version 1.0 in patients with measurable disease);  3. Rate of breast conserving surgery;  4. pCR rates in the breast and nodes;  5. pCR rates in the breast and in the breast and nodes by steroid hormone receptors status( positive or negative);  6. Objective response rates. |
| Study design | A non-comparative randomised multicentre phase IIb study;  Clinical trials identifier: NCT00450892. |
| GEICAM/2006-14 | |
| Author | E Alba 2014 |
| Participant | 102 patients enrolled (L: 52, T: 50)  Median (range) age: L: 48 (30-79), T: 48.5 (32-74)  Median follow up: unknown  Baseline comparability: balanced  Inclusion criteria: Female subjects with histologically proven stages I, II, III or inflammatory breast cancer (by breast core biopsy) and HER2-positive status, by local results, were included in this study. HER2 amplification was confirmed by Pathvysion FISH probes in a central laboratory, following the ASCO/CAP guidelines. Patients were eligible only if they were at least 18 years of age; had a Karnofsky performance status (PS) ≥ 80; had adequate bone marrow, liver, renal, and cardiac functions; and were treatment-naive. For women of childbearing age, a negative pregnancy test and use of adequate contraception were also required. Patients were excluded if they had the following: bilateral  invasive or metastatic breast cancer, a pre-existing neurotoxicity grade ≥ 2 (based on the National Cancer Institute-Common Terminology Criteria for adverse events version 3.0 (NCI-CTCAE v3.0) score system (Cancer Therapy Evaluation Program (CTEP), 2006)), a previous history of cancer other than cervical or non-melanoma skin cancer adequately treated, or other malignant tumours treated more than 10 years before the study entry; or any other severe or uncontrolled systemic disease. All patients provided written informed consent before study entry. |
| Intervention | **L arm:** (epirubicin 90 mg/m^2^ + cyclophosphamide 600 mg/m^2^) iv q21d * 4 cycles → docetaxel iv 100 mg/m^2^ q3w * 4 cycles + lapatinib po 1250 mg qd.  **T arm:** (epirubicin 90 mg/m^2^ + cyclophosphamide 600 mg/m^2^) iv q21d * 4 cycles → docetaxel iv 100 mg/m^2^ q3w * 4 cycles + trastuzumab iv 6 mg/kg (after a loading dose of 8 mg/kg) q21d. |
| Outcome | 1. pCR rate in the breast upon NAC treatment completion (assessed at surgery based on the Miller and Payne criteria.);  2. pCR rate in the breast (defined as the absence of any residual invasive tumour in the breast);  3. Breast and axilla pCR (defined as the absence of any residual invasive tumour in the breast and axilla at diagnosis in node-negative patients (grade 5-A) or in node-positive patients (grade 5-D));  4. Toxicity;  5. Adverse events(graded according to the NCI-CTCAE version 3.0.);  6. Rate of breast conservation;  7. Clinical response rates (defined as complete response (CR) + partial response (PR) was evaluated according to the Response Evaluation Criteria in Solid Tumours (RECIST) criteria after the fourth EC cycle and before surgery (upon NAC completion) using ultrasound, mammography, or MRI.). |
| Study design | A multicentre, open-label, randomised phase II trial;  Clinical trials identifier: NCT00841828 |
| NSABP B-41 | |
| Author | André Robidoux 2013 |
| Participant | 529 patients enrolled (T+L: 174, L: 174, T: 181)  Median (range) age: unknown  Median follow up: 1.9 years  Baseline comparability: balanced  Inclusion criteria: women aged 18 years or older with an ECOG performance status of 0 or 1 with operable HER2-positive breast cancer were eligible. breast tumor at least 2 cm or larger by palpation; clinical stage T2 to T3, N0 to N2a disease; no evidence of metastases; no history of previous invasive breast cancer; diagnosis by core needle biopsy; tumor with HER2 gene amplification by fluorescent in situ hybridisation (FISH) or chromogenic in situ hybridisation(CISH), or a strong positive (3+) staining score by immunohistochemistry; left ventricular ejection fraction(LVEF) assessment by multiple-gated acquisition scan or echocardiogram, of 50% or higher, regardless of the lower limit of normal of the assessing facility; no active cardiac disease or history of documented myocardial infarction, congestive heart failure, or cardiomyopathy. |
| Intervention | **T+L arm:** AC followed by paclitaxel plus trastuzumab plus lapatinib (trastuzumab 4 mg/kg IV followed by 2 mg/kg IV weekly beginning on day 1 of the first paclitaxel cycle until 1-7 days before surgery plus lapatinib 750 mg PO daily beginning on day 1 of the first paclitaxel cycle until 1 day before surgery)  **T arm:** AC then paclitaxel + trastuzumab (first dose: 4 mg/kg IV, subsequent doses: 2 mg/kg IV weekly beginning on day 1 of the first paclitaxel cycle until 1-7 days before surgery)  **L arm:** lapatinib 1250 mg PO daily beginning on day 1 of the first paclitaxel cycle until 1 day before surgery  AC followed by paclitaxel: four cycles of doxorubicin 60 mg/m2 and cyclophosphamide 600 mg/m2 intravenously on day 1 every 3 weeks, followed by four cycles of paclitaxel 80 mg/m2 intravenously on days 1, 8, and 15, every 4 weeks. |
| Outcome | 1. Pathological complete response in the breast (defined as the absence of any invasive component in the resected breast specimen.);  2. Pathological complete response in the breast and nodes (defined as absence of any invasive component in the resected breast specimen, and absence of cancer on haematoxylin and eosin evaluation of all resected lymph nodes after neoadjuvant therapy.);  3. The clinical complete response (after completion of the sequential neoadjuvant therapy regimen as assessed by physical examination);  4. Severe cardiac events (defined as definite or probable cardiac death, or New York Heart Association (NYHA) class III or IV congestive heart failure; and non-cardiac toxic effects.);  5. Adverse events (graded according to the National Cancer Institute (NCI) CTCAE v3.0. Information on grade 1 adverse events was not collected);  6. Rate of breast conserving surgery. |
| Study design | An open-label, randomised phase 3 trial;  Clinical trials identifier: NCT00486668. |
| LPT109096 | |
| Author | Frankie Ann Holmes 2013 |
| Participant | 100 patients enrolled (T+L: 33, L: 34, T: 33)  Median (range) age: T+L: 50 (28-66), L: 52 (25-67), T: 54 (21-67)  Median follow up: unknown  Baseline comparability: balanced  Inclusion criteria: ≥18 years of age; the presence of untreated, biopsy-proven, HER2-positive (immunohistochemistry [IHC] 3+ or fluorescence in situ hybridization [FISH] ratio >2.2), American Joint Commission on Cancer stage II or III invasive breast cancer; Eastern Cooperative Oncology Group (ECOG) performance status of 0 or 1; left ventricular ejection fraction ≥50% and within the institutional range of normal by echocardiogram or multi-gated acquisition scan; the ability to swallow and retain oral medication. |
| Intervention | **T+L arm:** trastuzumab (a loading dose of 4 milligrams [mg]/kilogram [kg] on Day 1, followed by a dose of 2 mg/kg on Day 1 of Week 2 and weekly thereafter) and lapatinib (1,000 mg PO daily, lapatinib 1000 mg daily, reduced to 750 mg daily during the 2-week run-in period and FEC, then increased to 1000 mg during paclitaxel (protocol amendment 3, as above).  **T arm:** trastuzumab alone (a loading dose of 4 milligrams [mg]/kilogram [kg] on Day 1, followed by a dose of 2 mg/kg on Day 1 of Week 2 and weekly thereafter).  **L arm:** lapatinib alone (1250 mg orally [PO] once daily [QD]). Participants were treated with lapatinib in a 2-week run-in period.  On Day 14, a second core needle biopsy was performed, followed by initiation of chemotherapy with 2 combination regimens of 4 cycles each (1 cycle=3 weeks): FEC75 (5-fluorouracil [5-FU] 500 mg/meters squared [m^2], epirubicin 75 mg/m^2, cyclophosphamide 500 mg/m^2 x 4 cycles on Day 1), then paclitaxel (80 mg/m^2 x 4 cycles on Day 1, Day 8, and Day 15) in combination with trastuzumab. |
| Outcome | 1. pCR ITT-E (defined as absence of invasive tumor in breast and lymph nodes);  2. pCR ITT (defined as absence of invasive tumor in breast and lymph nodes);  3. AE (National Cancer Institute Common Toxicity Criteria for AEs v3.0);  4. ORR (Response Evaluation Criteria in Solid Tumors 1.0.). |
| Study design | An open-label, randomized, phase II study;  Clinical trials identifier: NCT00524303. |

(Supplementary) Table 1: Details of included trials.
