# Supplementary material for: Lapatinib and lapatinib plus trastuzumab therapy versus trastuzumab therapy for HER2 positive breast cancer patients: an updated systematic review and meta-analysis
Source: Syst Rev. 2022 Dec 10;11:264. doi: 10.1186/s13643-022-02134-9 (PMC9738024; doi:10.1186/s13643-022-02134-9)
Supplement: Supplementary file 4 — Additional file 4: Table 2: Summary of meta-analysis and subgroup analysis results [file 13643_2022_2134_MOESM4_ESM.docx]

| Outcome | Pooled estimate / Subgroup | T+L vs. T | | | | | L vs. T | | | | |
| --- | --- | --- | --- | --- | --- | --- | --- | --- | --- | --- | --- |
|  |  | HR/RR 95%CI | *p* | I^2^ | Effect model | Superior arm | HR/RR 95%CI | *p* | I^2^ | Effect model | Superior arm |
| OS | Pooled estimate | HR: 0.84 [0.73, 0.97] | 0.02 | 0% | FEM | T+L | HR: 1.26 [1.08, 1.46] | 0.003 | 29% | FEM | T |
|  | I-III | / | / | / | / | / | HR: 1.05 [0.76, 1.47] | 0.76 | 48% | REM | T |
|  | MBC | / | / | / | / | / | HR: 1.40 [1.10, 1.80] | 0.007 | 0% | REM | T |
|  | Adjuvant therapy | HR: 0.87 [0.75, 1.01] | 0.06 | 0% | REM | T+L | / | / | / | / | / |
|  | Neoadjuvant therapy | HR: 0.65 [0.39, 1.06] | 0.09 | 10% | REM | T+L | HR: 0.85 [0.60, 1.20] | 0.36 | 0% | REM | L |
|  | Palliative therapy | / | / | / | / | / | HR: 1.40 [1.10, 1.80] | 0.007 | 0% | REM | T |
|  | HR+ | / | / | / | / | / | HR: 0.52 [0.22, 1.24] | 0.14 | 43% | REM | L |
|  | HR- | / | / | / | / | / | HR: 1.05 [0.65, 1.71] | 0.83 | 0% | REM | T |
| PFS | Pooled estimate | / | / | / | / | / | HR: 1.35 [1.11, 1.64] | 0.002 | 0% | FEM | T |
| DFS/EFS | Pooled estimate | HR: 0.89 [0.80, 0.98] | 0.02 | 9% | FEM | T+L | HR: 1.22 [1.05, 1.41] | 0.008 | 36% | FEM | T |
|  | Adjuvant therapy | HR: 0.90 [0.81, 1.00] | 0.04 | 0% | REM | T+L | / | / | / | / | / |
|  | Neoadjuvant therapy | HR: 0.67 [0.39, 1.17] | 0.16 | 33% | REM | T+L | / | / | / | / | / |
|  | HR+ | HR: 0.90 [0.78, 1.03] | 0.14 | 0% | REM | T+L | HR: 0.87 [0.58, 1.30] | 0.49 | 0% | REM | L |
|  | HR- | HR: 0.88 [0.76, 1.02] | 0.10 | 0% | REM | T+L | HR: 1.17 [0.82, 1.67] | 0.40 | 0% | REM | T |
| pCR (ypT0/is ypN0) | Pooled estimate | RR: 1.27 [1.13, 1.43] | <0.0001 | 14% | FEM | T+L | RR: 0.73 [0.65, 0.83] | <0.00001 | 22% | FEM | T |
|  | HR+ | RR: 1.15 [0.95, 1.38] | 0.15 | 0% | REM | T+L | RR: 0.82 [0.65, 1.04] | 0.10 | 0% | REM | T |
|  | HR- | RR: 1.22 [1.03, 1.45] | 0.02 | 0% | REM | T+L | RR: 0.83 [0.66, 1.04] | 0.10 | 0% | REM | T |
| pCR (ypT0/is ypN0/+) | Pooled estimate | RR: 1.31 [1.16, 1.49] | <0.0001 | 45% | FEM | T+L | RR: 0.79 [0.70, 0.89] | <0.0001 | 54% | FEM | T |
|  | HR+ | RR: 1.20 [0.95, 1.51] | 0.12 | 26% | REM | T+L | RR: 0.95 [0.76, 1.18] | 0.63 | 0% | REM | T |
|  | HR- | RR: 1.34 [1.11, 1.63] | 0.002 | 29% | REM | T+L | RR: 0.87 [0.71, 1.07] | 0.19 | 0% | REM | T |
| RFS | Pooled estimate | HR: 0.83 [0.72, 0.96] | 0.01 | 82% | FEM | T+L | / | / | / | / | / |
| ORR | Pooled estimate | RR: 1.02 [0.96, 1.09] | 0.53 | 56% | FEM | T+L | RR: 0.98 [0.93, 1.03] | 0.43 | 0% | FEM | T |
| DCR | Pooled estimate | / | / | / | / | / | RR: 0.96 [0.90, 1.01] | 0.13 | 0% | FEM | T |
| Rate of BCS | Pooled estimate | RR: 1.01 [0.88, 1.15] | 0.94 | 0% | FEM | T+L | RR: 0.94 [0.86, 1.04] | 0.24 | 14% | FEM | T |
| CHF | Pooled estimate | RR: 0.95 [0.73, 1.23] | 0.71 | 65% | FEM | T+L | RR: 0.89 [0.62, 1.28] | 0.54 | 45% | FEM | L |
|  | Adjuvant therapy | RR: 0.96 [0.52, 1.75] | 0.89 | 80% | REM | T+L | / | /73 | / | /2 | / |
|  | Neoadjuvant therapy | RR: 0.51 [0.03, 9.25] | 0.65 | 58% | REM | T+L | / | / | / | / | / |
| Decline of LVEF | Pooled estimate | RR: 0.82 [0.67, 1.01] | 0.06 | 52% | FEM | T+L | RR: 0.67 [0.50, 0.90] | 0.008 | 0% | FEM | L |
|  | Adjuvant therapy | RR: 0.80 [0.44, 1.44] | 0.45 | 87% | REM | T+L | / | / | / | / | / |
|  | Neoadjuvant therapy | RR: 0.57 [0.11, 2.97] | 0.50 | 0% | REM | T+L | / | / | / | / | / |
| Diarrhea | Pooled estimate | RR: 8.32 [6.49, 10.68] | <0.00001 | 57% | FEM | T | RR: 5.62 [4.41, 7.17] | <0.00001 | 81% | FEM | T |
|  | I-III | / | / | / | / | / | RR: 7.90 [5.88, 10.62] | <0.00001 | 0% | REM | T |
|  | MBC | / | / | / | / | / | RR: 0.99 [0.46, 2.15] | 0.99 | 36% | REM | L |
|  | Adjuvant therapy | RR: 6.62 [2.09, 20.94] | 0.001 | 94% | REM | T | / | / | / | / | / |
|  | Neoadjuvant therapy | RR: 10.56 [6.12, 18.23] | <0.00001 | 0% | REM | T | RR: 6.97 [4.46, 10.91] | <0.00001 | 0% | REM | T |
|  | Palliative therapy | / | / | / | / | / | RR: 0.99 [0.46, 2.15] | 0.99 | 36% | REM | L |
| Neutropenia | Pooled estimate | RR: 1.16 [0.86, 1.56] | 0.33 | 0% | FEM | T | RR: 0.99 [0.89, 1.09] | 0.82 | 59% | FEM | T |
|  | I-III | / | / | / | / | / | RR: 1.20 [0.77, 1.88] | 0.42 | 70% | REM | T |
|  | MBC | / | / | / | / | / | RR: 0.58 [0.27, 1.26] | 0.17 | 0% | REM | L |
|  | Neoadjuvant therapy | / | / | / | / | / | RR: 1.20 [0.77, 1.88] | 0.42 | 70% | REM | T |
|  | Palliative therapy | / | / | / | / | / | RR: 0.58 [0.27, 1.26] | 0.17 | 0% | REM | L |
| Fatigue | Pooled estimate | RR: 0.84 [0.42, 1.67] | 0.62 | 0% | FEM | T+L | RR: 1.44 [0.97, 2.11] | 0.07 | 0% | FEM | T |
| Rash/Skin toxicity | Pooled estimate | RR: 6.75 [4.66, 9.77] | <0.00001 | 0% | FEM | T | RR: 8.71 [5.64, 13.45] | <0.00001 | 0% | FEM | T |
|  | I-III | / | / | / | / | / | RR: 8.17 [4.82, 13.85] | <0.00001 | 8% | REM | T |
|  | MBC | / | / | / | / | / | RR: 4.86 [0.84, 28.03] | 0.08 | 0% | REM | T |
|  | Adjuvant therapy | RR: 7.49 [4.90, 11.45] | <0.00001 | 0% | REM | T | / | / | / | / | / |
|  | Neoadjuvant therapy | RR: 3.75 [1.68, 8.39] | 0.001 | 0% | REM | T | RR: 6.13 [2.85, 13.19] | <0.00001 | 5% | REM | T |
|  | Palliative therapy | / | / | / | / | / | RR: 4.86 [0.84, 28.03] | 0.08 | 0% | REM | T |
| Vomiting | Pooled estimate | RR: 2.17 [0.91, 5.19] | 0.08 | 0% | FEM | T | RR: 1.29 [0.69, 2.43] | 0.42 | 0% | FEM | T |
| Nausea | Pooled estimate | RR: 1.61 [0.64, 4.06] | 0.31 | 0% | FEM | T | RR: 1.02 [0.58, 1.80] | 0.94 | 0% | FEM | T |

(Supplementary) Table 2: Summary of meta-analysis and subgroup analysis results

HR: hazard ratio; RR: risk ratio; 95%CI: 95% confidence interval; OS: overall survival; T: trastuzumab; L: lapatinib; MBC: metastatic breast cancer; FEM: fixed effect model; REM: random effect model; HR+: hormone receptor positive; HR-: hormone receptor negative; OS: overall survival; PFS: progression free survival; DFS/EFS: disease free survival/event free survival; pCR: pathologic complete response; RFS: recurrence free survival; ORR: overall response rate; DCR: disease control rate; BCS: breast conserving surgery; CHF: congestive heart failure; LVEF: left ventricular ejection fraction.
